# Evolutionary impact assessment: accounting for evolutionary consequences of fishing in an ecosystem approach to fisheries management

**DOI:** 10.1111/faf.12007

**Published:** 2012-12-20

**Authors:** Ane T Laugen, Georg H Engelhard, Rebecca Whitlock, Robert Arlinghaus, Dorothy J Dankel, Erin S Dunlop, Anne M Eikeset, Katja Enberg, Christian Jørgensen, Shuichi Matsumura, Sébastien Nusslé, Davnah Urbach, Loїc Baulier, David S Boukal, Bruno Ernande, Fiona D Johnston, Fabian Mollet, Heidi Pardoe, Nina O Therkildsen, Silva Uusi-Heikkilä, Anssi Vainikka, Mikko Heino, Adriaan D Rijnsdorp, Ulf Dieckmann

**Affiliations:** 1 Swedish University of Agricultural Sciences, Department of Ecology,Box 7044, SE-75643, Uppsala, Sweden; 2 IFREMER, Laboratoire Ressources Halieutiques,Avenue du Général de Gaulle, F-14520, Port-en-Bessin, France; 3 Centre for Environment, Fisheries & Aquaculture Science (Cefas),Pakefield Road, Lowestoft, NR33 0HT, UK; 4 Evolution and Ecology Program, International Institute for Applied Systems Analysis (IIASA),Schlossplatz 1, A-2361, Laxenburg, Austria; 5 Hopkins Marine Station, Stanford University,120 Oceanview Blvd, Pacific Grove, CA, 93950, California, USA; 6 Finnish Game and Fisheries Research Institute,Itäinen Pitkäkatu 3, FI-20520, Turku, Finland; 7 Department of Biology and Ecology of Fishes, Leibniz-Institute of Freshwater Ecology and Inland Fisheries,Müggelseedamm 310, Berlin, 12587, Germany; 8 Department for Crop and Animal Sciences, Faculty of Agriculture and Horticulture, Humboldt-Universität zu Berlin,Philippstrasse 13, Haus 7, 10115, Berlin, Germany; 9 Institute of Marine Research,PO Box 1870, Nordnes, NO-5817, Bergen, Norway; 10 EvoFish Research Group, Department of Biology, University of Bergen,Box 7803, NO-5020, Bergen, Norway; 11 Aquatic Research and Development Section, Ontario Ministry of Natural Resources,300 Water Street, PO Box 7000, Peterborough, ON, Canada, K9J 8M5; 12 Department of Biology, Centre for Ecological and Evolutionary Synthesis (CEES), University of Oslo,PO Box 1066, Blindern, NO-0316, Oslo, Norway; 13 Computational Ecology Unit, Uni Research,PO Box 7810, NO-5020, Bergen, Norway; 14 Faculty of Applied Biological Sciences, Gifu University,Yanagido 1-1, Gifu, 501-1193, Japan; 15 Department of Ecology and Evolution, University of Lausanne,Biophore, CH-1015, Lausanne, Switzerland; 16 Conservation Biology, Bern University,Erlachstrasse 9a, CH-3012, Bern, Switzerland; 17 Department of Biological Sciences, Dartmouth College, The Class of 1978 Life Sciences Center,78 College Street, Hanover, NH, 03755, USA; 18 Fisheries and Aquatic Sciences Center, Agrocampus Ouest Centre de Rennes,65 rue de Saint Brieuc, CS 84215, F-35042, Rennes Cedex, France; 19 Department of Ecosystems Biology, Faculty of Science, University of South Bohemia,Branisovska 31, CZ-37005, České Budějovice, Czech Republic; 20 IFREMER, Laboratoire Ressources Halieutiques,150 quai Gambetta, BP 699, F-62321, Boulogne-sur-Mer, France; 21 Wageningen IMARES,Postbus 68, 1970, AB IJmuiden, The Netherlands; 22 Faculty of Life and Environmental Sciences, MARICE, University of Iceland,Askja, Sturlugata 7, 101, Reykjavik, Iceland; 23 Section for Population Ecology and Genetics, National Institute of Aquatic Resources, Technical University of Denmark,Vejlsøvej 39, DK-8600, Silkeborg, Denmark; 24 Division of Genetics and Physiology, Department of Biology, University of Turku,Pharmacity, FI-20014, Turku, Finland; 25 Department of Biology, University of Oulu,PO Box 3000, FI-90014, Oulu, Finland; 26 Swedish Board of Fisheries, Institute of Coastal Research,PO Box 109, SE-74222, Öregrund, Sweden; 27 Aquaculture and Fisheries Group, Department of Animal Sciences, Wageningen University and Research Centre,PO Box 338, 6700, Wageningen, The Netherlands

**Keywords:** Ecosystem approach to fisheries, ecosystem services, fisheries yield, fisheries-induced evolution, impact assessment, sustainable fisheries

## Abstract

Managing fisheries resources to maintain healthy ecosystems is one of the main goals of the ecosystem approach to fisheries (EAF). While a number of international treaties call for the implementation of EAF, there are still gaps in the underlying methodology. One aspect that has received substantial scientific attention recently is fisheries-induced evolution (FIE). Increasing evidence indicates that intensive fishing has the potential to exert strong directional selection on life-history traits, behaviour, physiology, and morphology of exploited fish. Of particular concern is that reversing evolutionary responses to fishing can be much more difficult than reversing demographic or phenotypically plastic responses. Furthermore, like climate change, multiple agents cause FIE, with effects accumulating over time. Consequently, FIE may alter the utility derived from fish stocks, which in turn can modify the monetary value living aquatic resources provide to society. Quantifying and predicting the evolutionary effects of fishing is therefore important for both ecological and economic reasons. An important reason this is not happening is the lack of an appropriate assessment framework. We therefore describe the evolutionary impact assessment (EvoIA) as a structured approach for assessing the evolutionary consequences of fishing and evaluating the predicted evolutionary outcomes of alternative management options. EvoIA can contribute to EAF by clarifying how evolution may alter stock properties and ecological relations, support the precautionary approach to fisheries management by addressing a previously overlooked source of uncertainty and risk, and thus contribute to sustainable fisheries.

**Table:** 

**Introduction**	**67**
**Processes in fisheries and their relation to FIE**	**68**
From fishing pressures to ecosystem dynamics	68
From ecosystem dynamics to ecosystem services	72
From ecosystem services to management measures	72
From management measures to fishing pressures	73
**Impacts of FIE on the utility of living aquatic resources**	**73**
Identifying ecosystem services	73
Valuating ecosystem services	75
Impact of FIE on the value of ecosystem services	75
Integrating values by utility	76
**Evolutionary impact assessment**	**77**
Types of evolutionary impact assessments	77
Quantifying the impacts of FIE	79
**Methods for evolutionary impact assessment**	**81**
Estimating the impact of fishing on traits	81
Demographic and evolutionary dynamics	83
Socioeconomic dynamics	84
Management-strategy evaluation	86
**Discussion**	**87**
**Acknowledgements**	**90**
**References**	**90**

## Introduction

Maintaining a healthy ecosystem while balancing competing interests of stakeholders is one of the main goals of the EAF (FAO 2003). Although there is an increasing scientific agreement that the EAF must encompass all aspects of an ecosystem, and a number of international treaties call for the implementation of the EAF, management of marine environments still largely concentrates on the yields extracted from harvestable resources. When management of these resources considers biological consequences of intense exploitation, the main focus usually lies on reducing the demographic and ecological effects of fishing. While this is undeniably important, ignoring other biological effects of fishing conflicts with the EAF. One such effect is temporal change in the life-history traits of exploited stocks, which many researchers have partially attributed to fisheries-induced evolution (FIE; Law and Grey 1989; Law 2000; Jørgensen *et al*. 2007; Allendorf *et al*. 2008). The most notable changes are shifts in maturation schedules towards earlier maturation at smaller sizes, which may negatively influence stock productivity and resilience to environmental change (Jørgensen *et al*. 2007). Despite mounting evidence for its prevalence, the ecological and socioeconomic consequences of FIE are not yet fully appreciated. Several studies have warned that ignoring FIE could result in negative impacts on the utility of exploited stocks, including reduced yield (Law and Grey 1989; Conover and Munch 2002; Matsumura *et al*. 2011), diminished genetic diversity (reviewed by Allendorf *et al*. 2008), and impaired recovery potential of stocks (de Roos *et al*. 2006; Walsh *et al*. 2006). FIE may therefore influence the profitability and viability of the fishing industry (Eikeset 2010), the quality of recreational fisheries (Matsumura *et al*. 2011), and certain aspects of coastal tourism (Jørgensen *et al*. 2007).

Assessments of exploited fish stocks are often highly uncertain (Cadrin and Pastoors 2008), and quantifying uncertainty in stock assessments has therefore been strongly advocated (e.g. Restrepo 1999). Given that ecologically driven uncertainty is large, it is not surprising that the considerable uncertainties associated with FIE are currently not accounted for in traditional forecasts of stock development. However, as stocks subject to heavy exploitation are expected to evolve over time (Jørgensen *et al*. 2007; Allendorf *et al*. 2008; Darimont *et al*. 2009), stock assessments and management advice ignoring evolutionary changes are likely to be less accurate than those accounting for the possibility of such changes. For example, estimated target or limit reference points may be biased when FIE is not accounted for (Hutchings 2009; ICES 2009; Enberg *et al*. 2010). Because of the complex nature of the ecological and evolutionary forces shaping populations, species, and ecosystems, fisheries scientists and managers need robust methods for evaluating the occurrence and extent of FIE and for assessing its effects on the monetary value that fish stocks provide to society. Furthermore, as life-history changes caused by FIE could be more difficult to reverse than plastic changes within the time periods relevant for fisheries management (Law and Grey 1989; de Roos *et al*. 2006; Conover *et al*. 2009; Enberg *et al*. 2009), it is vital to assess the likely impacts of FIE while mitigating actions can still be implemented in an effective manner. Owing to uncertainty about the rate and extent of FIE, its potential negative implications for the utility of stocks and its likely slow reversibility, incorporating FIE in stock assessments is mandated by the precautionary approach to sustainable fisheries management (FAO 2003).

Common-garden experiments have revealed rapid shifts in growth rate over relatively few generations in response to size-selective harvesting (Atlantic silversides, *Menidia menidia*; Conover and Munch 2002) and in age and size at maturation at experimentally increased mortality levels mimicking those imposed by commercial fishing (Trinidadian guppies, *Poecilila reticulata*; Reznick and Ghalambor 2005). Notwithstanding this experimental evidence and the theoretical expectations that genetic changes in heavily exploited populations are inevitable (Law and Grey 1989; Allendorf *et al*. 2008; Darimont *et al*. 2009), separating the effects of genetic processes and phenotypic plasticity on temporal trends in the wild is difficult because of the lack of controlled environmental conditions (Kuparinen and Merilä 2007). Detecting the presence of FIE and determining its relative importance is thus not straightforward. From a short-term perspective, quantifying the genetic and environmental causes underlying phenotypic trends may therefore seem unnecessary. After all, it is likely that a substantial proportion of the observed phenotypic changes are environmentally induced, and changing phenotypes will influence the utility of fish stocks irrespective of genetic or environmental origin. However, the long-term impacts on utility may differ greatly between environmentally and genetically induced changes in phenotypes. For example, if a fishing moratorium in a particular stock is implemented, plastic changes can be reversed relatively quickly. However, reversing genetic trends caused by high fishing mortality may take hundreds if not thousands of years of natural selection, which commonly is much weaker than human-induced selection (Law and Grey 1989; Darimont *et al*. 2009; Enberg *et al*. 2009; but see Edeline *et al*. 2007; Palkovacs *et al*. 2011 for claims that release from predation pressure can result in rapid genetically based phenotypic change).

Recent analyses of different fishery selectivity patterns can be used to formulate some general expectations for FIE in exploited stocks, and suggest ways to mitigate or reduce these impacts (Table 1). However, given the complexity of the interactions between historical, current, and predicted natural and harvest-induced selection, simple rules of thumb are not reliable in all situations. Thus, we urgently need more stock-specific models accounting for the eco-evolutionary dynamics of exploitation. While accounting for genetic changes in stock properties is warranted under the EAF paradigm, to date, the estimation of FIE and its effects on utility has occurred only sporadically, mostly in academic settings, and without a collection of appropriate analytical tools. The evolutionary impact assessment (EvoIA) introduced by Jørgensen *et al*. (2007) is meant to serve as a component of the management-strategy evaluation (MSE) framework in fisheries (Smith *et al*. 1999). It aims at moving one step further towards bridging the gap between current fisheries management and the EAF by accounting for an underappreciated aspect of the biological consequences of fishing. Using a variety of methods, EvoIA aims to quantify the potential costs of FIE and to evaluate the evolutionary consequences of alternative management options for mitigating potential undesired impacts.

**Table 1:** Expectations for FIE of life-history traits and possible mitigation for two different selectivity patterns. A sigmoidal selectivity curve represents a scenario in which there is a minimum-size limit for harvested fish and harvesting targets all fish above this minimum-size limit (e.g. many types of trawls). A dome-shaped curve may have both maximum- and minimum-size limits so that both large and small fish are protected, but is not constrained to be symmetrical (e.g. many types of gillnets)

Selectivity pattern	Expectations	Possible mitigative actions
Sigmoidal	Size-refuge of small fish increases the advantage of staying small, leading to evolution towards smaller sizes and younger ages even at low fishing mortality (Boukal *et al*. 2008; Dunlop *et al*. 2009a,2009b; Enberg *et al*. 2009; Jørgensen *et al*. 2009; Kuparinen *et al*. 2009; Mollet *et al*. 2010; Box 2)	Increase the minimum-size limit, that is, protect a larger proportion of the size spectrum
	The stronger the fishing pressure, the larger the evolutionary response (Dunlop *et al*. 2009a,2009b; Enberg *et al*. 2009; Jørgensen *et al*. 2009; Kuparinen *et al*. 2009; Mollet *et al*. 2010; Matsumura *et al*. 2011; Box 2)	Force a dome-shaped selectivity pattern by introducing a maximum-size limit (not possible for all types of fishing gear)
	Harvesting mature individuals selects for later maturation at larger sizes, whereas harvesting only immature individuals or both mature and immature individuals selects for earlier maturation at smaller sizes (Ernande *et al*. 2004)	Reduce fishing mortality to precautionary levels
	Feeding-ground reserve (marine protected area) favours delayed maturation, spawning-ground reserve favours earlier maturation (Dunlop *et al*. 2009b)	Implement well-tailored marine protected areas or seasonal moratoria
	FIE of growth rate depends on the difference between minimum-size limit and size at maturation; minimum-size limits below size at maturation increases growth rate with the opposite effect for higher minimum-size limits (Boukal *et al*. 2008; Dunlop *et al*. 2009a)	
	High evolutionarily stable yield can be achieved only with very low harvest rates (Jørgensen *et al*. 2009; Mollet *et al*. 2010; Box 2)	
	Recovery of genetic traits to pre-harvest levels is slow compared to the speed of FIE (Enberg *et al*. 2009)	
Dome-shaped	If gear captures mostly smaller fish, that is, for highly asymmetrical dome shapes: we expect shifts towards later maturation at larger sizes (Boukal *et al*. 2008; Kuparinen *et al*. 2009)	Adjust the width and the position of the harvestable size range (harvestable-slot length limits); e.g. adjust the mesh size of gillnets or implement combination of minimum-length and maximum-length limits for recreational fisheries
	If gear protects both small and large fish: the intensity of harvesting vs. the intensity of natural selection towards increased size and higher fecundity determine the evolutionary response (Boukal *et al*. 2008; Jørgensen *et al*. 2009).	Reduce fishing mortality to precautionary levels
	At high fishing mortality, few individuals escape the harvestable size range leading to earlier maturation at smaller sizes (Jørgensen *et al*. 2009).	
	If less-intense fishing reduces the chances of being caught until growing larger than the maximum-size limit, growing to a large size to increase fecundity may be adaptive, depending on the relative strengths of the selection pressures (Boukal *et al*. 2008; Jørgensen *et al*. 2009; Mollet *et al*. 2010; Box 2).	
	Implementing harvest-slot length limits under positively size-selective fishing with the lower bound of the slot set larger than the maturation size, reduces selection on maturation size and age, and leads to positive selection on immature growth rate (Matsumura *et al*. 2011)	
	Evolutionarily stable yield can be obtained under higher fishing mortality than for sigmoidal selectivity (Jørgensen *et al*. 2009; Mollet *et al*. 2010; Box 2)	
	Maximum evolutionarily sustainable yield depends on time horizon (Mollet *et al*. 2010; Box 2)	

**Figure 1:**
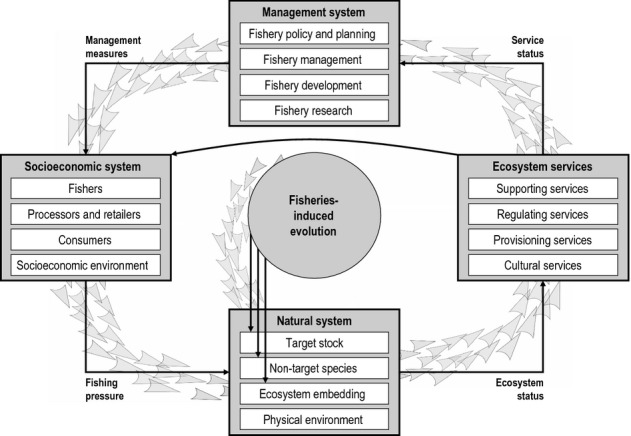
Schematic illustration of the interactions among the main components of a fishery system. The thin black arrows represent direct interactions, whereas the grey triangular arrows illustrate how the direct effects of fisheries-induced evolution (FIE) on the natural system cascade through the fishery system, affecting fishery management and the socioeconomic system through their impacts on ecosystem services (see Fig. 2 for an example detailing such a cascading effect).

**Figure 2:**
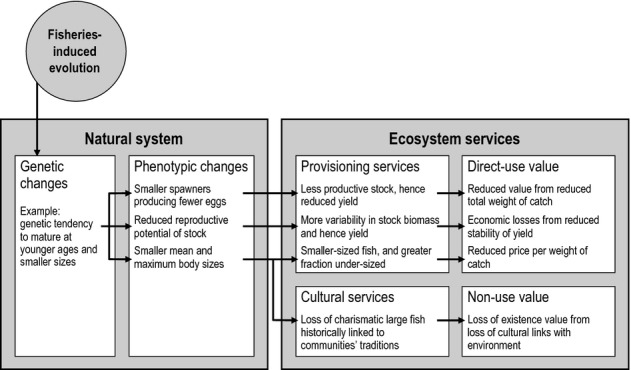
Example of the cascading effects of fisheries-induced evolution (FIE) on ecosystem services and their values. This illustrates how the effects of FIE on a single trait of one component of the natural system (reduced age and size at maturation in the target stock) may impact two ecosystem services (provisioning and cultural services) and associated socioeconomic values (direct-use value and non-use value). Specific applications of the evolutionary impact assessment (EvoIA) framework may capture fewer or more ecosystem services, and fewer or more linkages may connect these with associated socioeconomic values. This illustration is therefore by no means exhaustive: fishing may also cause the evolution of other traits and have a variety of indirect effects on different ecosystem services and associated socioeconomic values.

**Figure 3:**
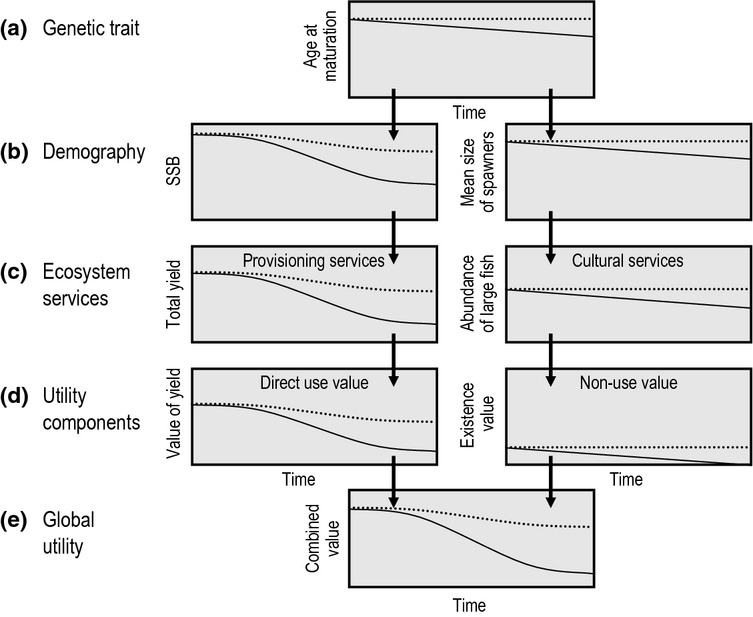
Schematic illustration of a hypothetical *retrospective* evolutionary impact assessment aiming to quantify the consequences of past fisheries-induced evolution (FIE) from the genetic trait to a global utility function. All curves, therefore, show effects of changes in the genetic component of the trait in question. The assessment compares time series of quantities of interest from an evolutionary scenario (continuous lines) with those from a non-evolutionary scenario (dashed lines) given a particular fishing regime. (a) This example focuses on FIE in a stock's average age at maturation and assumes that FIE causes fish to mature at earlier ages and smaller sizes. (b) In the evolutionary scenario, fishing results in more rapid decreases in spawning-stock biomass (SSB) and in the average body size of spawners. (c) This will influence ecosystem services: provisioning services decline because of a more strongly reduced yield, and cultural services decline, for example, because of the loss of desirable large fish. (d) This implies secondary effects on the associated socioeconomic values or utility components: direct-use values are diminished because of a less valuable total yield, and non-use values are diminished because of the loss of existence value. (e) The loss of values from provisioning and cultural services can be assessed jointly, in terms of a global utility function, which is found to decline more strongly as a result of FIE. Note that although FIE may often lead to earlier maturation at smaller sizes, as shown in this example, under particular circumstances, it may result in delayed maturation.

**Figure 4:**
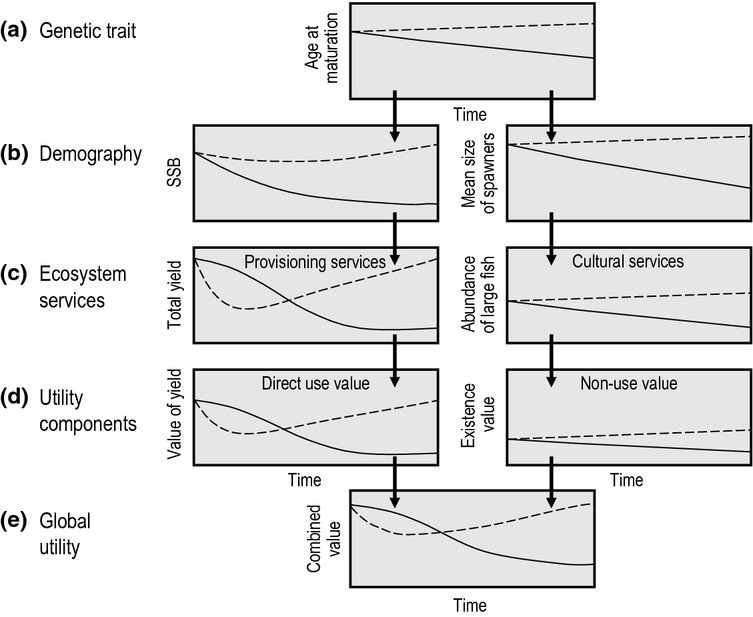
Schematic illustration of a hypothetical *prospective* evolutionary impact assessment aiming to evaluate two alternative management regimes while accounting for the potential effects of fisheries-induced evolution (FIE). All curves, therefore, show effects of changes in the genetic component of the trait in question. The assessment compares time series of quantities of interest between a status-quo management regime (continuous lines) and an alternative management regime aiming to mitigate FIE by changing fishing selectivity (dashed lines). (a) The status-quo regime is assumed to cause a continual decline of the stock's mean age and size at maturation, whereas the alternative regime is assumed to enable an evolutionary recovery. (b) The status-quo regime implies more severe phenotypic effects – a steadily declining spawning-stock biomass (SSB) and a diminishing average body size of spawners – than the alternative regime, with the latter leading to recovery of SSB and increasing fish size. (c) This has consequences for ecosystem services: provisioning services monotonically decline with yield under the status-quo regime, whereas a steep initial decline is followed by recovery under the alternative regime. Similar conclusions apply to cultural services affected by the loss or preservation of large desirable fish. (d) This implies secondary effects on the associated socioeconomic values or utility components. (e) While the resultant global utility is found to decline monotonically under the status-quo regime, it recovers under the alternative regime. Note that although FIE may often lead earlier maturation at smaller size, as shown in this example, under particular circumstances, it may result in delayed maturation.

**Figure 5:**
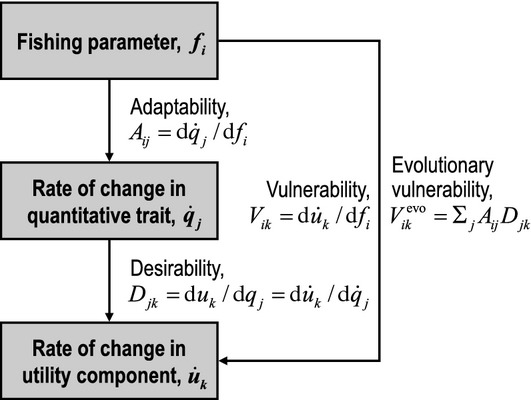
Four sensitivity measures of particular relevance in evolutionary impact assessment (EvoIA). The adaptability *A*_*ij*_ measures the sensitivity with which a change in the fishing parameter *f*_*i*_ alters the evolutionary rate 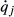 of the quantitative trait *q*_*j*_. The desirability *D*_*jk*_ measures the sensitivity with which a change in the quantitative trait *q*_*j*_ alters the utility component *u*_*k*_ (according to the chain rule, this is equivalent to the sensitivity with which a change in the evolutionary rate 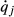 of the quantitative trait *q*_*j*_ alters the rate of change 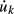 in the utility component *u*_*k*_). The vulnerability *V*_*ik*_ measures the sensitivity with which a change in the fishing parameter *f*_*i*_ alters the rate of change 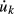 in the utility component *u*_*k*_. The evolutionary vulnerability 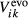 measures the part of the vulnerability *V*_*ik*_ that is caused by FIE. EvoIAs can estimate the matrices *A*,*D*,*V* and *V*^evo^.

**Figure 6:**
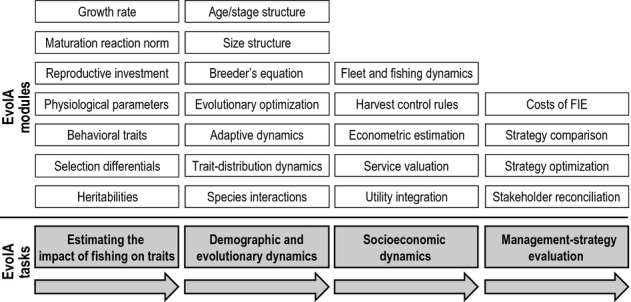
Main types of building blocks in an evolutionary impact assessment (EvoIA). When devising a specific EvoIA, practitioners can go through up to four tasks (grey boxes). These are best carried out in an order as indicated by the arrows, although not every EvoIA will necessarily address all four tasks. For carrying out each task, different modules are available (white boxes). While not all modules have to be used in each EvoIA, different modules may need to be combined to address a task. The modules listed here are not intended to be exhaustive. Methods associated with each module are mentioned in the main text.

**Figure 7:**
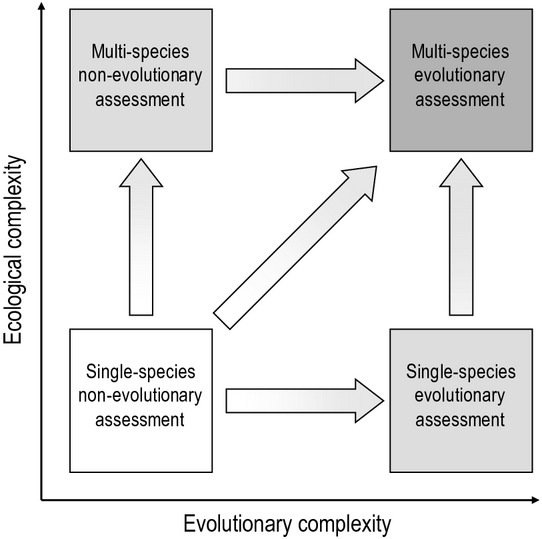
Evolutionary impact assessment (EvoIA) facilitates accounting for two major dimensions of complexity confronting modern fisheries management – evolutionary complexity and ecological complexity. Current single-species management (bottom-left box) incorporates variable degrees of ecological detail, but omits interspecific interactions (top-left box) and evolutionary impacts (bottom-right box). The vertical arrow on the left represents ongoing developments towards multispecies or ecosystem-based approaches to fisheries management, whereas the horizontal arrow at the bottom represents developments towards single-species EvoIA. The top-right box represents an EvoIA that explicitly accounts for the evolutionary consequences of fishing in an ecosystem approach to fisheries management.

Here, we expand upon the concept of EvoIA introduced by Jørgensen *et al*. (2007). We start by giving an overview of fishery systems and how FIE may influence their various components (section *Processes in fisheries and their relation to FIE*; Fig. 1). We then outline how an EvoIA can help quantify the effects of FIE on the different components of a stock's utility (sections *Impacts of FIE on the utility of living aquatic resources* and *Evolutionary impact assessment*; Figs 2–5). We also explain how to carry out an EvoIA in practice, highlight which methods are available for that purpose, and point to studies that have used these methods to quantify FIE (section *Methods for evolutionary impact assessment*; Fig. 6). Finally, we describe how an EvoIA may support the transition from traditional fisheries management to implementing the EAF (section *Discussion*; Fig. 7). Key terms and abbreviations are explained in Box 1.

**Box 1. Glossary**

- **Discount rate:** An interest rate used to convert the value of a sum of money due in the future relative to its worth today. The discount rate reflects the opportunity cost of investing money in a particular action or project, given that it could have earned interest elsewhere.
- **Eco-evolutionary dynamics:** Feedback between ecological and evolutionary dynamics in which ecological change leads to (rapid) evolutionary change and microevolutionary change influences ecological processes (Pelletier *et al*. 2009).
- **Ecosystem approach to fisheries:** The goals of the EAF are ‘to balance diverse societal objectives, by taking into account the knowledge and uncertainties about biotic, abiotic, and human components of ecosystems and their interactions and applying an integrated approach to fisheries within ecologically meaningful boundaries’ (FAO 2003). Extending the conventional fisheries-management paradigm, ‘the approach thus intends to foster the use of existing management frameworks, improving their implementation and reinforcing their ecological relevance, and will contribute significantly to achieving sustainable development’ (Garcia and Cochrane 2005).
- **Ecosystem services:** ‘The benefits people obtain from ecosystems’ (Millennium Ecosystem Assessment 2003). *Supporting services* are the basis for the three following categories of ecosystem services and benefit humans through fundamental long-term ecological processes, including nutrient cycling and primary production, and may thus be directly or indirectly affected by FIE through changes to ecological and genetic processes. *Regulating services* benefit humans through ecosystem regulation such as climate and disease regulation or water purification and water-quality control (e.g. water clarity), which may be impacted if FIE changes trophic interactions, size structures, or migration distances. *Provisioning services* benefit humans through tangible products such as fisheries yields, recreational fishing experiences, and economic rents and are likely to be modified by FIE through changes in the characteristics and demography of stocks and the dynamics of communities. *Cultural services* benefit humans through the values ecosystems offer for education, recreation, spiritual enrichment, and aesthetics, which may all be affected if FIE occurs.
- **Fisheries-induced evolution:** ‘Genetic change in a population, with fishing serving as the driving force of evolution’ (ICES 2007). Includes both neutral and adaptive genetic changes.
- **Fishery system:** The entire system in which a fishery operates, including subsystems such as the socioeconomic system of fishers, fishing companies, and the sellers and buyers of fish products; the natural system of target and non-target species and their ecosystem and environmental settings; the ecosystem services provided to humankind; and the management system consisting of fishery management, planning and policy, fishery development, and fishery research (Charles 2001).
- **Net present value:** ‘The difference between the present value of a future flow of profits arising from a project and the capital cost of the project’ (Bannock *et al*. 2003).
- **Opportunity cost:** ‘The value of that which must be given up to acquire or achieve something’ (Bannock *et al*. 2003).
- **Precautionary approach:** Principle 15 of Agenda 21 agreed on at the Earth Summit meeting at Rio de Janeiro in 1992: ‘In order to protect the environment, the precautionary approach shall be widely applied by States according to their capabilities. Where there are threats of serious or irreversible damage, lack of full scientific certainty shall not be used as a reason for postponing cost-effective measures to prevent environmental degradation’ (UN 1992).
- **Selection differential:** The difference between the mean trait value of a population and the mean of the individuals selected to be parents of the next generation.
- **Selection pressure:** A general term describing the extent to which reproductive success varies across the current phenotypes in a population. Over time across generations, selection pressure is expected to lead to a change in the composition of genetic traits in a population, provided the phenotypes under selection have a heritable component.
- **Stocks and populations:** A stock is usually a management unit and can include one or several populations, or only part of a population. A population is a biological/evolutionary unit often defined as a collection of interbreeding individuals in a given area and can belong to several stocks or form part of one stock. When assessing the presence and importance of FIE, knowledge about the evolutionary units present in a particular area is crucial, as growth trajectories and maturation schedules and thereby the impact of FIE may differ between units.
- **Trait:** Here, we define a trait as a character of interest for fisheries management, for example, growth rate, age or size at maturation. While the expression of these quantitative traits is dependent on a multitude of other quantitative traits, they are particularly interesting because of their influence on the utility of fish stocks. Moreover, they are characters that are relatively easy to estimate from the type of data available to fisheries scientists. The main goal of EvoIA is to quantify how the genetic component of traits changes with selection pressures. Thus, unless otherwise stated, ‘trait’ refers to the estimated genetic component of a quantitative character, often with an unknown molecular-genetic basis.
- **Utility:** ‘The pleasure or satisfaction derived by an individual from being in a particular situation or from consuming goods and services’ (Bannock *et al*. 2003). Utility can be, but need not be, expressed in monetary units.
- **Utility components:** Various attributes of a system from which utility is derived, contributing to the total utility associated with the system. Stock abundance, biodiversity, employment, profit, and yield are important utility components associated with fisheries. Stakeholders often differ in the utility they ascribe to these various components.
- **Utility function:** ‘A mathematical representation of consumer preferences for goods and services’ (Calhoun 2002). More specifically, utility functions describe how the value stakeholders attribute to utility components varies with the status of these components and how the utility derived from these individual components is combined into a measure of a system's total utility.

## Processes in fisheries and their relation to FIE

Fisheries-induced evolution may affect all parts of a fishery system: (i) the natural system, including the target stock, non-target species, and the surrounding ecosystem and its physical environment, (ii) the resulting ecosystem services generated by targeted fish stocks, (iii) the management system, and (iv) the socioeconomic system (Fig. 1). Each of these subsystems can be described at multiple levels of complexity (Charles 2001), such as single-species or multispecies ecology, single-component or multicomponent ecosystem services, single-agency or multiagency management, and single-fleet or multifleet fisheries. Because these subsystems interact, the impacts of FIE may result in cascades of indirect effects rippling through a fishery system (Fig. 1; Jackson *et al*. 2001).

### From fishing pressures to ecosystem dynamics

Fishing impacts the natural system in several ways. First are the demographic effects on target stocks (Beverton and Holt 1957) such as reduced abundance and biomass (Hutchings and Myers 1994; Toresen and Østvedt 2000), truncated age and size-structure (Jørgensen 1990), and modified geographical distribution (Overholtz 2002). Demographic changes may have consequences for the genetic composition of stocks including altered population- genetic subdivision and erosion of genetic diversity (Allendorf *et al*. 2008). Second are the effects on trait expression through phenotypic plasticity. Reduced abundances may lead to increased *per capita* resource availability and thus to faster individual growth and reduced age at maturation (Jørgensen 1990; Engelhard and Heino 2004), the latter of which might change maternal-effect contributions and average fecundity (Venturelli *et al*. 2009; Arlinghaus *et al*. 2010). Exposure to fishing may result in behavioural gear avoidance (Wohlfarth *et al*. 1975; Raat 1985; Askey *et al*. 2006; Rijnsdorp *et al*. 2008) and modified migration routes (Prodanov *et al*. 1995; Jørgensen *et al*. 2008; Parsons 2011), and truncated population structures can alter size-based behavioural interactions within and among species (Huse *et al*. 2002). Third are the adaptive genetic consequences of fishing (Heino and Godø 2002). Fishing pressure may selectively favour earlier maturation at smaller size (Jørgensen *et al*. 2007), change the shape of reaction norms for maturation (Christensen and Andersen 2011; Marty *et al*. 2011), alter growth rates (Sinclair *et al*. 2002; Edeline *et al*. 2007; Swain *et al*. 2007; Nusslé *et al*. 2008; Enberg *et al*. 2012), and change reproductive investment (Yoneda and Wright 2004; Rijnsdorp *et al*. 2005). It may also affect behavioural and physiological traits through selection for less vulnerable or bold individuals (Heino and Godø 2002; Biro and Post 2008; Uusi-Heikkilä *et al*. 2008; Philipp *et al*. 2009) or by disrupting hermaphroditism (Sattar *et al*. 2008) or sexual selection (Hutchings and Rowe 2008; Urbach and Cotton 2008). Other possible adaptive changes include altered spawning migrations and geographical distributions (Jørgensen *et al*. 2008; Thériault *et al*. 2008). Fourth are the effects that go beyond the target stock. By-catch of other species is often inevitable (Goldsworthy *et al*. 2001), causing changes in demography, phenotypic plasticity, and genetic characteristics of non-target species. Competitors, predators, and prey of target species can be affected (Hiddink *et al*. 2006) when the properties of target stocks change. The effects of fishing and possibly also FIE can further induce trophic cascades (Frank *et al*. 2005) and trigger ecosystem-level regime shifts affecting nutrient cycling and predator–prey interactions (Daskalov *et al*. 2007; Palkovacs *et al*. 2012). Fifth are the impacts of fishing on the physical environment such as pollution and seafloor habitat destruction (Watling and Norse 1998). Traditional approaches to fisheries management tend to focus on demographic effects on target species. However, the EAF necessitates increased awareness of all impacts of fishing. EvoIA is designed to address the evolutionary dimension of this broadening focus.

### From ecosystem dynamics to ecosystem services

Living aquatic resources provide a variety of ecosystem services to society and stakeholders (Daily 1997). There are different classifications of these services, each fulfilling a different purpose (Costanza 2008). In the context of an EvoIA, we suggest using the four categories of ecosystem services considered in the Millennium Ecosystem Assessment (2003). Their definitions are described in Box 1, and their socioeconomic valuation, including utility components and utility functions, is described in more detail in the section *Impacts of FIE on the utility of living aquatic resources* below.

The status of an ecosystem determines the status of the associated ecosystem services (Fig. 1), which may be changed by FIE in several ways. FIE typically causes earlier maturation, in some cases also increased reproductive investment, and may therefore lead to a decreased average size at age after maturation. As a consequence, the biomass caught at a certain fishing-mortality rate decreases under constant recruitment (Matsumura *et al*. 2011). Furthermore, FIE towards gear avoidance reduces catch per unit effort or requires continuous development of gears and fishing techniques (Rijnsdorp *et al*. 2008; Philipp *et al*. 2009). FIE towards diminished genetic diversity may impair a stock's resilience to environmental perturbations and thereby threaten its stability (Hsieh *et al*. 2010). By changing properties of stocks such as their size structure, FIE could also promote or even trigger ecological regime shifts in food webs and thus undermine associated regulating services (Anderson *et al*. 2008). Finally, FIE might impact an ecosystem's cultural value through the genetic alteration of life histories or behaviour. All these changes feed through to the utility that society derives from an exploited ecosystem.

### From ecosystem services to management measures

The management of aquatic ecosystems involves many stakeholders (Hilborn 2007). Under the EAF paradigm, fisheries management should consider all stakeholder interests when identifying and implementing measures for improving the benefits of fishing that might matter to a society. Together with the demands of stakeholders, the status of the ecosystem services should determine appropriate management measures (Fig. 1). The management subsystem broadly involves fishery research, identification of suitable management measures and policy making, as well as planning, implementation, and development of the fishing industry, including processing and trade. These tasks in general, and decisions about management measures in particular, imply trade-offs between different stakeholder interests (Wattage *et al*. 2005). Because FIE may affect ecosystem services as outlined above, its existence and extent are likely to influence which management measures are adopted, and should therefore also influence fishery data collection and research. EvoIA enables fisheries managers to account for FIE in their decision-making by evaluating the ecological and socioeconomic effects of FIE, and thus highlights opportunities for mitigation. While the management of other natural resources could also indirectly be affected by FIE, here we focus on the effects of FIE on fisheries management.

### From management measures to fishing pressures

Aided by regulation and enforcement, management measures such as input controls (e.g. effort limitation such as seasonal closures or number of hooks allowed) and output controls (e.g. catch limitations such as total allowable catches or minimum landing sizes) are intended to alter fishing pressure. However, several factors within the socioeconomic subsystem may shape realized fishing pressures because they influence the decisions taken by individual fishers about their fishing activities (Salas and Gaertner 2004; Johnston *et al*. 2010). Employment and profit maximization (BenDor *et al*. 2009) and the opportunity cost of fishing (i.e. the cost of forgone activities) are often key considerations. Community traditions, within-community competition, habits, subsidies, and market demands also influence the dynamics of effort, labour, capital, technology, and activity of a fishing fleet and thus the total investment, geographic and seasonal distribution, and stock-specific targeting of fishing efforts (Branch *et al*. 2006; Rijnsdorp *et al*. 2008). In recreational fisheries, non-catch-related motives are additional factors determining the activity of a population of fishers (Johnston *et al*. 2010). The socioeconomic subsystem also comprises the consumers of fishing products. Consumer preferences define demand, which in turn is mediated by processors and retailers, and which ultimately determines economic incentives for fishers. Certification schemes designed to alter consumer preferences may create incentives for fishers and managers to bring their practices into better compliance with the certificate's requirements (Kaiser and Edwards-Jones 2006). A greater awareness of the potentially adverse effects of FIE among fishers, certification organizations, and consumers could help divert fishing pressure from stocks that have been identified as particularly vulnerable to FIE.

## Impacts of FIE on the utility of living aquatic resources

Organizations in charge of fisheries management are often expected to evaluate the link between biological and socioeconomic aspects of fishing (Charles 2001); in many countries, this is even required by law. Nevertheless, explicitly incorporating social objectives into fisheries policy is often neglected (Symes and Phillipson 2009). As a small contribution towards addressing this issue, EvoIA is designed to quantify both the ecological and the socioeconomic impacts of FIE, in terms of its potential consequences for the utility of exploited stocks and associated ecosystem components. This requires attributing values to different ecosystem services (Fig. 2) and quantifying how FIE changes the utility of fish stocks. Such a task consists of four steps: (i) identifying ecosystem services provided by living aquatic resources potentially affected by FIE, (ii) valuating these ecosystem services, (iii) identifying the impacts of FIE on the value of ecosystem services, and (iv) integrating these values in a global utility function. Below, we describe each of these steps. While a comprehensive EvoIA covers all four steps, EvoIAs may also comprise just a subset of these steps.

### Identifying ecosystem services

A fishery's utility represents the total benefit stakeholders derive from engaging in fishing. The attributes of fisheries and ecosystems from which stakeholders derive total utility are known as utility components (Walters and Martell 2004). These include properties such as yield and its variability, genetic diversity, recreational quality involving both catch (e.g. size of trophy fish) and non-catch (e.g. aesthetics) components of the experience, fisheries-related employment and ecosystem functioning. Some stakeholders value undisturbed stocks and ecosystems and thus prefer full protection of aquatic biodiversity. However, such objectives usually conflict with the aim of maximizing fisheries profits or employment, which are the main goals of other stakeholders (Hilborn 2007). Traditionally, fisheries-management objectives have been tailored towards fishers as the principal stakeholders (Wattage *et al*. 2005; Hilborn 2007). The primary focus of these stakeholders is generally maximizing yields or employment (Larkin 1977) in the fishing industry or maximizing social yield (Johnston *et al*. 2010) in recreational fisheries. Other utility components, such as preservation of genetic diversity, natural population structure, or ecological interactions, have only recently received attention. The intangible nature of these latter utility components makes them more difficult to measure and valuate (Balmford *et al*. 2002), because they are not captured by conventional market-based economic activity. However, the need to account for utility components other than those reflecting direct use is widely recognized and drives the current move from single-species fisheries management to an ecosystem approach (Francis *et al*. 2007).

Utility functions quantify how utility components contribute to a fishery's total utility according to their values as perceived subjectively by stakeholders. Given the often-disparate interests and objectives among stakeholders (Wattage *et al*. 2005) in terms of outcomes and combinations of utility components (Bannock *et al*. 2003), their utility functions are likely to differ. For example, a commercial fisher's utility function is mainly driven by the maximization of net revenue (BenDor *et al*. 2009), while a conservationist might emphasize the preservation of a species' role in an ecosystem more or less undisturbed by human action. Inputs into fishery utility functions tend to focus on provisioning services and can include quantities such as annual catch, average size of fish caught, economic revenue, and catch stability. Additional, sometimes implicit, inputs may be measures of ecosystem preservation, fisheries-related employment, or fisheries profits (Law 2000; Wattage *et al*. 2005; Hard *et al*. 2008). Realistically, we expect discussions about the evolutionary impacts of fishing to center around provisioning services in general and fisheries yields in particular. Therefore, the potential impacts of FIE on provisioning services will probably be the initial focus of an EvoIA, even though the effects on other ecosystem services should eventually also be quantified and addressed. Additionally, because supporting and regulating services cannot always be easily distinguished (Hein *et al*. 2006), we combine these two service categories and hereafter refer to regulating services as comprising all contributions of living aquatic resources to ecosystem structure, function, and resilience.

### Valuating ecosystem services

Methods for valuating ecosystem services are described, for example, by Costanza *et al*. (1997) and Wallace (2007). For the purpose of this article, we distinguish four value categories. *Direct-use value* comes from the direct utilization of living aquatic resources, includes consumptive use values (e.g. harvest) and non-consumptive use values (e.g. recreational catch-and-release fishing or scuba-diving), and arises from provisioning and cultural services (Fig. 2). *Indirect-use value* comes from the indirect benefits that living aquatic resources provide in terms of promoting ecosystem stability and resilience (e.g. through the maintenance of trophic structures) and primarily arises from regulating services. *Option value* comes from the potential future use of living aquatic resources or related ecosystem components such as yet to be discovered resources with medicinal or industrial use and can arise from all ecosystem services. *Non-use value* comes from attributes inherent to a living aquatic resource or related ecosystem components that are not of direct or indirect use to members of society but still provide value to stakeholders (Fig. 2). This includes intrinsic value (based on the utility derived from knowing that something like a species or a natural gene pool exists), altruistic value (based on utility derived from knowing that somebody else benefits from using nature), and bequest value (based on utility gained from future improvements in the well-being of one's descendants). Non-use values only arise from cultural services and ethics, and are the most difficult services to quantify (Hein *et al*. 2006). While it is popular, and sometimes convenient, to express utilities in a common monetary unit, it should be borne in mind that this is by no means necessary. Elaborate methodologies such as random-choice theory (McFadden 1974; Hensher *et al*. 2005) exist for quantifying monetary as well as non-monetary utility components based on statistical information about stakeholder choices and preferences collected, for example, through questionnaires. For calibrated statistical choice models in the context of fisheries research, see, for example, Aas *et al*. (2000) or Dorow *et al*. (2010).

### Impact of FIE on the value of ecosystem services

Evolutionary impacts on the direct-use value of living aquatic resources occur when changes in life-history traits attributed to FIE positively or negatively affect stock productivity (Enberg *et al*. 2010). Changes in stock productivity can for example be expected from earlier maturation, increased reproductive investment, and lower growth rates. For instance, North Sea plaice (*Pleuronectes platessa*, Pleuronectidae) now mature at younger ages and smaller sizes than in the past (Grift *et al*. 2003), cod (*Gadus morhua*, Gadidae) in the North Sea and west of Scotland are now more fecund than 30 years ago (Yoneda and Wright 2004), and the Gulf of Saint Lawrence cod have shown likely fisheries-induced changes in growth rates (Swain *et al*. 2007). Such impacts might interact in nonlinear ways: although earlier maturation may cause a larger fraction of a population to become adult, this adult fraction might in total become less fecund because of diminished size at age or reduced offspring survival resulting from smaller average egg size.

Indirect-use value may be affected through changes in trophic interactions: if a predatory fish species becomes smaller, it may shift to smaller prey, which in turn could imply altered ecosystem functioning through a trophic cascade (Jackson *et al*. 2001). While the structural and functional changes that occurred in the Scotian Shelf ecosystem (Frank *et al*. 2011) have not been directly linked to FIE (but see Shackell *et al*. 2010), it provides a good example of altered indirect-use value through reduced body size, reduced biomass, altered species composition, and reduced individual condition in several fish species (Choi *et al*. 2004).

A stock's option value and non-use value may also diminish as a result of FIE (Fig. 2). For instance, because the reversal of FIE-triggered changes in life-history traits is predicted to be slow once high fishing pressure has ceased (Law and Grey 1989; de Roos *et al*. 2006; Dunlop *et al*. 2009a), the recovery of total stock biomass to original levels is delayed compared to a situation in which FIE has not occurred (Enberg *et al*. 2009). Note, however, that while the model of Enberg *et al*. (2009) predicts that recovery of total biomass is delayed when FIE occurs, it also predicts that spawning-stock biomass and recruitment recover faster after FIE. Option value may also be reduced if the systematic removal of larger fish increases variance in yield (van Kooten *et al*. 2010) and leads to FIE towards smaller fish, potentially bringing about an alternative stable state, after which the ecosystem continues to be dominated by smaller-sized and thus less valuable fish (Persson *et al*. 2007). Further, if FIE decreases genetic diversity, populations may become less resistant to environmental stress, which in turn may reduce option value and non-use value. All these changes might impair a wider set of non-use values for non-fishing members of society. For example, one non-use value likely to diminish through FIE is the satisfaction of knowing about the existence of a healthy fish community; some stakeholders may dislike genetic alterations of fish stocks because this conflicts with existence, altruistic, or bequest values.

### Integrating values by utility

Integrating the values of the various utility components into a global utility function occurs at two levels. First, stakeholders decide – implicitly or explicitly – how to integrate the utility components important to them into an integrated utility function representing their interests. Second, managers decide how to combine these utility functions across all stakeholders into one global function on which management decisions can be based. Constructing a global utility function – particularly at the management level, but also at the stakeholder level – usually implies prioritizing utility components and thus involves addressing the trade-offs among them (Walters and Martell 2004; Wattage *et al*. 2005). For example, intensive size-selective exploitation might bring about a short-term gain in one particular ecosystem service (e.g. direct-use value from provisioning services of the exploited fish stock) while at the same time eroding other ecosystem services (e.g. indirect-use value from regulating services). These trade-offs are partly shaped by the time frames at which stakeholders value the different services (Walters and Martell 2004; Carpenter *et al*. 2007; see below). In the simplest case, global utility functions are specified as weighted sums of utility components, with weights reflecting the prioritization of different objectives (Dankel *et al*. 2007). In more complex scenarios, global utility may be expressed through nonlinear functions (Johnston *et al*. 2010) to account for interactions among different utility components. While specifying a global utility function is not a prerequisite for implementing an EvoIA, it is desirable for a transparent and quantifiable approach.

Evaluating changes in utility components must account for time, as most stakeholders tend to value future utility less than present utility. A discount rate is therefore often used to convert the value of gains or losses in the future to net present value, figuratively trading goods and services across time (Carpenter *et al*. 2007). High discount rates imply a preference for realizing gains in the present and delaying costs to the future. Although FIE can occur surprisingly rapidly (Jørgensen *et al*. 2007; see Andersen and Brander 2009 for an alternative perspective on speed), the time over which FIE unfolds might still cover decades. This is significantly longer than the time frames often considered in conventional fisheries management, so that the choice of discount rate is bound to have large effects on EvoIAs. In particular the relative importance of plastic and genetic trait changes and thus expected impacts on yield over time are strongly affected by discount rates. Use of discount rates is most easily defensible when considering purely economic values, an approach that has *de facto* dominated decision-making in traditional fisheries management. However, from a conservation point of view, one might argue that a positive discount rate is not justified, as intrinsic values or the rights of future generations must not be discounted. Ultimately, this involves moral and ethical debates that need to be settled outside the scientific domain.

The second step, that is, deciding how to integrate the utility functions of all stakeholders to obtain one global utility function determining management decisions, is also largely a political choice. Decision-makers must determine which utility components, global utility function, and discount rate best reflect the collective interests of stakeholders in their constituency. Naively, weighting the utility functions of different stakeholder groups by their prevalence in the population would seem the most democratic approach. In practice, however, such an approach may be problematic, both because it might fail to protect the legitimate interests of minorities, and because the interests articulated by stakeholders are not always based on sufficient information and rational evaluation. Therefore, the integration of stakeholder interests is typically at the discretion of politicians and managers.

Negotiating and deciding on a global utility function is an inherently complex process. Currently, stakeholder involvement in fisheries management remains the exception rather than the rule, and when negotiations occur, quantitative specifications of utility components are often lacking. Nevertheless, ultimately only the quantification of stakeholder utilities and the mutual understanding of the used criteria can enable a maximally informed debate. When the interests of stakeholders and the decisions of politicians are articulated quantitatively, the political process of reconciling divergent interests in terms of a global utility function can become more transparent.

## Evolutionary impact assessment

An EvoIA typically include two major steps: the assessment of how fishing practices may induce genetic changes in exploited stocks and the examination of how such evolutionary changes may alter the utility components through which living aquatic resources and their ecosystems provide value to stakeholders and society.

While fishing in some cases has been shown to reduce effective population size and thereby diminish general genetic diversity (Hauser *et al*. 2002; Hutchinson *et al*. 2003; but see, e.g. Poulsen *et al*. 2006; Therkildsen *et al*. 2010 for examples of large effective population sizes despite intensive fishing), we will in the following sections focus on genetic changes in individual traits, because of their stronger effects on productivity and management. In principle, however, an EvoIA could be used to quantify the effects of both neutral and adaptive evolution imposed through fishing.

In the simplest case, EvoIA can quantify the effects of FIE on a single trait and a single utility component such as biomass yield for a single stakeholder (Law and Grey 1989; Vainikka and Hyvärinen 2012). However, including multiple traits and utility components for multiple stakeholders may be required for a more realistic assessment. Ideally, EvoIA is based on a global utility function reflecting overall management objectives developed through stakeholder involvement. However, an EvoIA can also deal with separate utility components, which may be desirable to expose the trade-offs between conflicting objectives (Walters and Martell 2004), and with multiple global utility functions that individually reflect the disparate interests of stakeholders.

### Types of evolutionary impact assessments

Two types of EvoIA help address distinct challenges arising from FIE: (i) quantification of the losses or gains in utility that may result from FIE and (ii) evaluation of alternative management regimes while accounting for the potential effects of FIE. The first type, illustrated in Fig. 3, quantifies the consequences of FIE by including or removing the effect of FIE in a simulated fishery system. To evaluate alternative scenarios, statistical or process-based models or both are needed: an evolutionary scenario allowing the genetic component of traits to change in response to fishing, and a corresponding non-evolutionary scenario in which the genetic component of traits are kept constant over time. Being otherwise identical, the two scenarios may also track the effects of changing traits on the demography of the target stock and other ecosystem elements and address how these demographic changes impact relevant ecosystem services and utility components (for an application to recovery dynamics, see Enberg *et al*. 2009). A further step could integrate utility components into a global utility function. In the hypothetical example illustrated in Fig. 3, this integration (i.e. the step from Fig. 3d to e) includes the direct-use value from provisioning services and the non-use value from cultural services. The example shows how a relatively small change in a genetic trait may sometimes result in a significant negative impact on global utility. However, in other cases, FIE may have little negative impact on utility, or may even improve global utility.

The second type of EvoIA, illustrated in Fig. 4, evaluates the outcome of two or more alternative management options while accounting for the potential occurrence of FIE. Once again, this requires statistical or process-based models or both. The different model scenarios describe the different management options under consideration, but are otherwise identical in quantifying the expected genetic and phenotypic changes, demographic effects, impacts on ecosystem services, and alteration of utility components (for examples of analyses of the consequences of different fishing gears for life-history evolution and yield, see Jørgensen *et al*. 2009; Mollet 2010). A dome-shaped selection pattern protecting larger fish may, for instance, have evolutionary effects opposite to those of the typically implemented sigmoid selection pattern focusing on larger fish (Jørgensen *et al*. 2009; Mollet 2010; Matsumura *et al*. 2011). Although leaving large fish may result in short-term losses of yield (see Arlinghaus *et al*. 2010 for an example in which protecting the large fish maintained and sometimes even increased yield relative to exploitation using minimum-length limits), there may be long-term gains in yield. Using a global utility function, the total socioeconomic consequences expected to result under alternative scenarios can be assessed and compared. The hypothetical example in Fig. 4 illustrates such a comparison. In the first management regime, sustained moderate overfishing causes continual trait evolution, steadily declining yields, and hence reduced direct-use values (decreasing total catches) and lessened non-use values (loss of culturally important charismatic large fish). In the alternative management regime, relaxed fishing pressure (assuming absence of genetic constraints) not only results in a different direction of trait evolution, but also (after an initial strong decline in yield) eventually results in higher yields and larger fish (Matsumura *et al*. 2011), leading to enhanced direct-use and non-use values.

Despite efforts to predict the direction of FIE for different kinds of selection regimes (e.g. Table 1), producing general predictions and advice for mitigation across species, stocks, traits, and fishing regimes is difficult. Therefore, EvoIAs need to address case studies that analyse the evolutionary impacts of a particular fishing regime on a particular stock's ecology. It is therefore necessary to calibrate models to empirical data. The retrospective part of an EvoIA then uses the results of the data analysis and a comparison between non-evolutionary and evolutionary versions of the model to better understand past FIE (if it occurred), its impact on past stock dynamics, and the consequences of past management measures. When the fraction of the observed phenotypic change attributable to FIE cannot be clearly identified, some simplifying assumptions are needed. For instance, assuming that the entire observed phenotypic change is attributable to FIE, even when an environmental component is likely but unknown, could provide the basis for analysing a worst-case scenario (with regard to the induced evolutionary changes, not necessarily in terms of other consequences of fishing). Such an analysis could reveal the maximum amount of genetic change that can be expected from a particular fishing regime. By contrast, the aim of the prospective part of an EvolA is to forecast the future extent and impact of FIE. Such forecasts can be used for evaluating different management measures, such as spatial effort allocation or use of fishing gears with different selective properties that may minimize unwanted FIE (Law and Rowell 1993; Hutchings 2009; Jørgensen *et al*. 2009; Mollet 2010). Comprehensive EvoIAs are likely to use these two types of analysis in combination, first to assess the extent to which FIE is relevant for a stock's dynamics and then to evaluate which measures are most advisable for managing the stock in the light of the impacts caused by FIE.

### Quantifying the impacts of FIE

To quantify the impacts of fishing on evolvable traits and utility components, three groups of quantities and their relationships must be analysed. First are fishing parameters, such as fishing mortality or minimum landing size, which characterize quantitative features of a fishing regime. Other fishing parameters of interest might describe fishing effort or quantitative features of fishing gears, marine reserves, or seasonal closures. Second are quantitative traits, measuring a stock's evolvable characteristics. These include heritable characteristics describing maturation schedules, growth trajectories, and reproduction schemes. While it is common to focus on stock-level mean genetic values of such quantitative traits, measures of diversity, such as trait variances and genetic correlations among traits, can (and ultimately should) also be considered. When evaluating the causal relationships between these two groups of quantities, it is crucial to recognize that fishing parameters do not change quantitative traits directly. Instead, they alter the selection pressures operating on phenotypes and thus the expected rates of evolutionary change. When these rates are integrated over a given time period, they yield the magnitude by which the quantitative trait will change in response to the altered fishing parameters. Because selection pressures may differ over the lifetime of individuals, an assessment of the relative strength of larval, juvenile, and adult selection pressures is warranted (Johnson *et al*. 2011). Additionally, any temporal variation in fishing selectivity (Kendall *et al*. 2009) should be accounted for. Third are the utility components described in section *Impacts of FIE on the utility of living aquatic resources*. The proposed EvoIA framework can theoretically accommodate any number of fishing parameters, quantitative traits, and utility components. Obviously, the more ingredients are investigated at once, the more complex an EvoIA will become, which may lead to overly demanding analyses and difficult interpretation.

EvoIAs sometimes have to examine scenarios that involve relatively large departures from a fishery system's current state. Such departures may originate from various drivers, including the demographic, plastic, evolutionary, ecosystem, and physical impacts of fishing, as well as external drivers of the fishery system. Large departures can occur when the magnitude of driver change is large, or when analysing relatively long time periods. To describe the resulting impacts, models then have to account for nonlinearities in the relationships among and within the fishery subsystems (Fig. 1). While quantifying nonlinearities may be required for accurate assessments beyond a short time period, reliable estimation of nonlinear relationships from empirical data is often difficult. Therefore, basing EvoIAs on simpler linear analyses may be of interest. Such analyses are powerful as long as a system is not forced too far away from its current state.

Linear impact analyses are based on sensitivity measures. Once a sensitivity measure has been estimated, the impacts of changes in a fishing parameter are obtained simply by multiplying this measure with the magnitude of change in the causative parameter and, where the result is a rate, by multiplying it with the duration of the considered time period. If changes in several fishing parameters are considered at once, their aggregated impact is obtained by summing their individual impacts. The following four sensitivity measures (Fig. 5) may be of particular relevance in EvoIAs. *Adaptability* is known in ecology as a system's ability to cope with uncertainty and perturbations (Conrad 1983). In the context of EvoIA, we define it more specifically as the sensitivity with which a change in a fishing parameter alters a quantitative trait's evolutionary rate. When the absolute value of adaptability is high, the genetic component of the quantitative trait quickly changes according to the considered change in fishing. Positive (negative) adaptability means that the quantitative trait's evolutionary rate increases (decreases) in response to an increase in the considered fishing parameter. The change in the quantitative trait's evolutionary rate might originate from direct selection pressure imposed by fishing, or indirectly, through genetic covariance or pleiotropy with other evolving traits. *Desirability* is the sensitivity with which a changing quantitative trait alters a utility component. When the absolute value of desirability is high, the utility component is strongly influenced by the quantitative trait so that, and this is mathematically equivalent, the rate of change in this utility component is strongly influenced by the rate of change in the quantitative trait. Positive (negative) desirability means that the utility component increases (decreases) as the considered trait value increases. *Vulnerability* is the sensitivity with which a change in a fishing parameter alters the rate of change in a utility component. When the absolute value of vulnerability is high, the utility component quickly changes in response to the considered change in fishing. Positive (negative) vulnerability means that the rate of change in the utility component increases (decreases) in response to an increase in the considered fishing parameter.

It is critical to appreciate that a fishing parameter's impact on a utility component often has nothing to do with FIE. We therefore introduce a fourth quantity, *evolutionary vulnerability*, as the sensitivity with which a change in a fishing parameter alters the rate of change in a utility component through FIE. Following the multivariate chain rule of calculus, we define this as the product of adaptability and desirability summed over all considered quantitative traits (Fig. 5). We here define traits as the genetic component of the life-history traits in question, so that the trait changes reflect genetic and not plastic changes. This definition implies that evolutionary vulnerability only concerns changes in the rate of change of a utility component that originate through evolutionary changes in the considered traits. In other words, evolutionary vulnerability ignores the effects of altered fishing parameters on utility components not mediated by genetic changes in life-history traits. When the absolute value of evolutionary vulnerability is high, the rate of change in utility component through FIE in response to the considered change in fishing is high. Positive (negative) evolutionary vulnerability means that the utility component increases (decreases) through FIE in response to an increase in the considered fishing parameter. The difference between vulnerability and evolutionary vulnerability describes non-evolutionary changes in utility caused by fishing, and the ratio of evolutionary vulnerability and vulnerability describes the proportion of vulnerability caused by FIE. Assessing and comparing these two measures thus yields important insights into a stock's vulnerability to fishing. In an EvoIA, large negative evolutionary vulnerabilities ought to be a cause for concern: these occur when changed fishing patterns cause rapid FIE that is detrimental to utility.

## Methods for evolutionary impact assessment

EvoIAs requires methods that enable practitioners to estimate trait values and their trends, to study the demographic and evolutionary dynamics of populations and communities, to account for the socioeconomic objectives of stakeholders, and to quantify a fishery's utility accordingly. On this basis, practitioners can evaluate the evolutionary impact that alternative management measures may have on exploited stocks. Therefore, the EvoIA approach encourages integrating methods that until now have often been used in isolation. To facilitate a structured approach, we now distinguish between four tasks addressed by EvoIAs and review the corresponding methods. These tasks and methods serve as building blocks for assembling specific EvoIAs and are illustrated in Fig. 6. The combination of the methods we present here is highly flexible, and they can and should be tailored to the needs of each particular fishery system, as has recently been done for North Sea plaice (Box 2).

**Box EvoIA example: North Sea plaice**

The EvoIA of North Sea plaice by Mollet *et al*. (2010) is among the very first of its kind. The authors explored the impact of FIE on the productivity of plaice using an eco-genetic individual-based model by comparing different management scenarios with and without an evolutionary response. They showed that under a status-quo scenario in which larger plaice are more likely to be caught than smaller ones, plaice evolve towards smaller size at age, earlier maturation, and higher reproductive investment (see also Grift *et al*. 2003). Their model predicts that as a consequence, the biological reference points of maximum sustainable yield (MSY) and corresponding fishing mortality (F_MSY_) should be reduced relative to the current reference points for this stock, which ignore FIE. This is because the estimated optimal fishing mortality when FIE is ignored (‘static’ F_MSY_) is well above the evolutionarily optimal fishing mortality (‘evolutionary’ F_MSY_). Hence, even if the stock would be fished at the currently estimated ‘static’ F_MSY_, this mortality would still be too high and decrease the future yield. The currently advised reference points can therefore not be considered sustainable.

Mollet *et al*. (2010) also show that the evolutionary response can be reversed, by changing fishing effort and size selectivity. This would require a dome-shaped exploitation pattern through which plaice of intermediate size are most likely to be caught and not just the smallest but also the largest fish escape the mortality window. In the case of North Sea plaice, managers have the option to apply such a dome-shaped exploitation pattern by influencing the spatiotemporal behaviour of the trawling fleet, as plaice are distributed in space and time according to their size, with larger individuals feeding further offshore; only for reproduction, all size classes are encountered on the spawning grounds (Rijnsdorp *et al*. 2012). In the short term, a dome-shaped exploitation pattern would imply a loss in yield, as the largest fish are not caught, but this would trade off against the long-term loss that would otherwise ensue because of evolution resulting in smaller-sized fish. The optimal levels of effort and selectivity depend on the time horizon considered: on a timescale of years to a few decades, a strategy targeting larger fish gives more yield, but on a multidecadal to centennial timescale, the long-term evolutionary impact becomes increasingly important.

**Figure:**
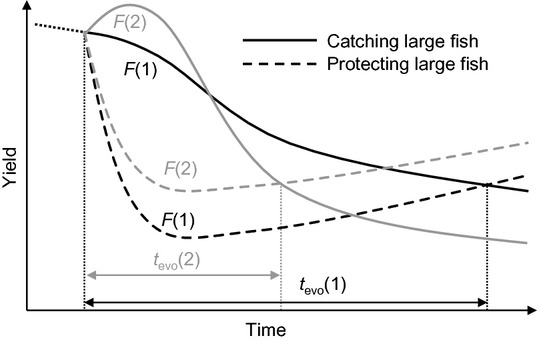
Long-term trends in predicted North Sea plaice yield under moderate [*F*(1)] and high [*F*(2)] fishing-mortality levels and under two patterns of size selectivity: a sigmoidal selectivity pattern through which larger fish are most likely to be caught (solid lines) and a dome-shaped selectivity pattern through which intermediate fish are most likely to be caught with the largest escaping (dashed lines). *t*_evo_ represents the time span until the short-term gain in yield from catching large fish falls below the long-term evolutionary gain from protecting them. This time span is longer under moderate fishing mortality than under high fishing mortality, *t*_evo_(1) > *t*_evo_(2).

## Estimating the impact of fishing on traits

A range of statistical methods is available for quantifying changes in life-history traits and other traits over time, and for determining the relative importance of phenotypic plasticity and evolution in generating observed changes. Broadly speaking, these methods – which have been applied to patterns of growth, maturation, and reproduction – examine the plausibility of an evolutionary interpretation of observed phenotypic changes by (i) analysing environmental variables, (ii) estimating selection pressures, and (iii) examining multiple stocks. The three paragraphs below outline these approaches in turn.

Some methods control for environmental variance in life-history traits by including relevant additional explanatory variables in the fitted statistical models, thus aiming to remove the effects of phenotypic plasticity from genetic trends. While the removal of all other known effects will never be possible, residual year or cohort effects may indicate evolutionary change. For instance, the estimation of probabilistic maturation reaction norms (PMRNs) was developed to disentangle genetic and environmentally induced changes in age and size at maturation, by accounting for growth variation (Dieckmann and Heino 2007). Recent experimental evaluations, however, call for caution in the interpretation, as the method may overestimate or underestimate genetic influence on changes in PMRNs, depending on environmental and genetic circumstances (Kinnison *et al*. 2011; Uusi-Heikkilä *et al*. 2011). The PMRN approach has been extended to control for other factors influencing maturation, such as condition (Grift *et al*. 2007; Mollet *et al*. 2007; Vainikka *et al*. 2009; Uusi-Heikkilä *et al*. 2011). Other authors have controlled for the effects of temperature-dependent and density-dependent growth to identify residual changes in growth rates that may be ascribed to evolution (Swain *et al*. 2007). Corresponding methods have also been developed for addressing potential evolution in reproductive investment (Rijnsdorp *et al*. 2005; Baulier 2009). Directly or indirectly, the aforementioned methods are all based on the concept of reaction norms (e.g. Reznick 1993) and describe how the translation of genotypes into phenotypes is changed by environmental factors.

Although the statistical methods mentioned above can be applied using data commonly available from harvested fish, it remains impossible to separate genetic responses from all potential plastic responses in life-history traits for most wild fish stocks (Dieckmann and Heino 2007; Kinnison *et al*. 2011; Kuparinen *et al*. 2011; Uusi-Heikkilä *et al*. 2011). This is because a number of genetic and environmental processes – such as temporal collinearity, phenotypic correlations, genetic covariance, genotype-by-environment interactions and counter-gradient variation – can confound phenotypic patterns that might be attributed to genetic responses. Estimating selection differentials (Law and Rowell 1993; Olsen and Moland 2011) therefore adds important knowledge about the relationship among life histories, fishing patterns, and the resultant expected strengths of selection on relevant quantitative traits, and thereby enables a critical evaluation of hypothesized evolutionary responses to fishing. While fitness itself is difficult to estimate in marine systems, proxies such as viability or fecundity are often used. Assuming that selection acts only through viability and if sufficiently detailed data are available describing the composition of cohorts with respect to a trait of interest, selection differentials can be estimated directly. For example, Nusslé *et al*. (2008) measured selection differentials on growth by comparing the growth of fish from the same cohort, caught at different ages. In anadromous fish such as salmonids, catch and escapement data from rivers may be used to estimate selection differentials for size and age at maturation (Kendall *et al*. 2009) or size at age (Saura *et al*. 2010). However, selection seldom acts only through viability. Thus, when fecundity selection is involved, or when cohorts are insufficiently sampled, the estimation of selection differentials requires model-based full–life cycle analyses of the fitness consequences of trait changes (e.g. Arlinghaus *et al*. 2009; Matsumura *et al*. 2011). Together with the estimated heritability of traits, selection differentials enable quantifying responses to selection through the breeder's equation.

Regardless of the nature of the phenotypic trends in commercial fish stocks, an additional challenge in EvoIA is to link the observed trends to fishing pressure. This is directly related to the general problem of inferring causation from correlation in insufficiently controlled settings. One way to alleviate – albeit not remove – this problem is to include multiple fish stocks in a single analysis. For example, one can test whether fishing pressure is correlated with rates of trait changes across multiple fish stocks, as suggested by Sharpe and Hendry (2009). However, when applying this idea, it must be kept in mind that different life histories may respond evolutionarily to the same fishing pressure in ways that can differ not only quantitatively (i.e. in terms of the rate of evolutionary change), but also qualitatively (i.e. in terms of the direction of evolutionary change) and temporally (i.e. in terms of how best to align the time series of fishing pressure with the time series of traits). Consequently, a weak correlation between fishing pressure and the rates of trait changes does not carry a strong implication, whereas a strong correlation could indeed strengthen the interpretation that the observed changes are caused by fishing.

An additional complication arises when fisheries are targeting mixed assemblages of fish from several different evolutionary units, such as in the migrating Atlantic herring (Ruzzante *et al*. 2006) or the North Sea cod (Holmes *et al*. 2008). Thus, if the resolution of the available fisheries and survey data does not reflect the genetic population structure in targeted stocks, it will not be possible to disentangle within-population changes from shifting migration patterns of different population components. One of the high-priority tasks must therefore be that data collection on commercially exploited stocks is biologically meaningful and is reflecting the existing genetic structure. As long as the genetic substructure of many stocks is still unknown and structured-population data is still lacking, estimates of FIE from the existing data must incorporate this uncertainty, and a precautionary approach is warranted as much as ever (Hutchinson 2008).

## Demographic and evolutionary dynamics

EvoIAs typically require examination of the demography and evolution of populations and, ideally, ecological communities (Fig. 6). We can broadly categorize corresponding models as being either statistical or process-based; these alternative approaches offer different strengths and limitations. First, to describe demographic or evolutionary changes in a population retrospectively, statistical models use time as one explanatory variable among others. By contrast, process-based models successively update a system's changing state variables through time via difference or differential equations. External drivers, such as relevant environmental factors, are represented by explanatory variables in statistical models and by changing parameters in process-based models. Because all effectors in process-based models are known, such models are useful to study complex temporal trends, especially when interactions among the drivers of such trends are nonlinear. The findings of such analyses may be helpful when interpreting the outcome in statistical analyses. Second, for assessing the costs of FIE, process-based models make it easy to ‘switch off’ evolution, so that the impact of a management measure on utility can be compared between an evolving and a non-evolving population (Enberg *et al*. 2009; Eikeset 2010; Mollet 2010; Box 2). This allows isolation of genetically mediated changes in utility. If statistical models are used for population projections, year or cohort effects attributed to evolution can be explicitly removed to predict behaviour in the absence of evolution (Heino *et al*. 2002). Third, although statistical methods can be used for population projections (by extrapolating time series and the impacts of drivers), process-based models usually offer greater capacity and flexibility in predicting a system's behaviour in the future or under alternative management regimes. Fourth, to evaluate alternative management measures, extrapolations based on statistical models are likely to be of limited use, especially when such measures are expected to take a system far away from its current state. Moreover, process-based models facilitate modelling a broad range of uncertainties in fishery systems, by accounting for observed or anticipated patterns of fluctuations and trends in external drivers. Thus, prospective EvoIAs will rely primarily on process-based models.

Models used for EvoIA can also be classified according to the variables structuring the demographic component of stock dynamics. In the context of modelling FIE, researchers have used age-structured models (e.g. Law and Grey 1989; Law and Rowell 1993; Gårdmark *et al*. 2003; Bradshaw *et al*. 2007; Eldridge 2007; Arlinghaus *et al*. 2009) and continuously size-structured models (Ernande *et al*. 2004; de Roos *et al*. 2006; Morita and Fukuwaka 2006; Dunlop *et al*. 2009b,2009a; Enberg *et al*. 2009; Vainikka and Hyvärinen 2012). Stage structure is useful for distinguishing between mature and immature individuals or to describe spatially segregated fishing grounds. However, many practical questions associated with EvoIA requires, for example, distinguishing between mature fish of different sizes. Models based on stage structure alone are therefore often insufficient for detailed comparisons with data, because of their overly simplified demography.

A further distinction among process-based models arises from methods used for quantifying the effects of selection, and thus for describing the evolutionary component of stock dynamics (Fig. 6). In modelling FIE, researchers have estimated selection differentials (Law and Rowell 1993), selection responses based on the breeder's equation of quantitative genetics theory (de Roos *et al*. 2006; Hilborn and Minte-Vera 2008; Nusslé *et al*. 2008; Andersen and Brander 2009; Arlinghaus *et al*. 2009), evolutionary outcomes based on evolutionary optimization models and ESS theory (Law and Grey 1989; Heino 1998; Jørgensen *et al*. 2009), selection responses based on the canonical equation of adaptive dynamics theory (Gårdmark *et al*. 2003; Ernande *et al*. 2004; de Roos *et al*. 2006), and finally, selection responses based on modelling the dynamics of the full trait distributions of quantitative traits (Baskett *et al*. 2005; Dunlop *et al*. 2007, 2009a,2009b; Arlinghaus *et al*. 2009; Enberg *et al*. 2009; Okamoto *et al*. 2009; Matsumura *et al*. 2011).

Depending on the objectives of a specific EvoIA, a population's demographic and evolutionary dynamics may best be described by different combinations of the alternative model choices described above. Nevertheless, one type of models, coined ‘eco-genetic’ models (Dunlop *et al*. 2009a), offers a particularly suitable process-based modelling framework for use in EvoIA. Eco-genetic models account for continuous size structure and describe the full trait distributions of quantitative traits. They integrate quantitative genetic detail with ecological detail, enable a tighter coupling to empirical data than many traditional models, and allow the prediction of evolutionary rates, transients, and endpoints (Dunlop *et al*. 2007, 2009a,2009b; Thériault *et al*. 2008; Enberg *et al*. 2009; Okamoto *et al*. 2009; Wang and Höök 2009). The recent scientific focus on eco-evolutionary dynamics leaves little doubt that phenotypic changes, whether they are plastic or genetic in nature, may have far-reaching effects on food webs and ecosystems. Because the eco-genetic models described above are difficult to extend to multispecies cases, including interactions and feedbacks between species in EvoIAs typically rely on simpler kinds of quantitative modelling (Gårdmark *et al*. 2003; Matsuda and Abrams 2004).

## Socioeconomic dynamics

EvoIAs need to evaluate the socioeconomic implications of the impacts of fishing on ecosystem services and utility values. Usually, this can be achieved by coupling a biological model of a stock to a socioeconomic model describing the utility components stakeholders derive from that stock. The complexity of the latter models may range from relatively simple, focusing on a small set of readily quantifiable utility components, such as yield or profit (e.g. Dankel 2009; Eikeset 2010; Mollet 2010; Box 2), to more comprehensive models using a global utility function and as many utility components as possible (Johnston *et al*. 2010). Additional utility components may, for instance, characterize the quality of the fishing experience or describe the benefits and costs that fishing activities imply for society. Examples of the former are quantitative measures of catch stability, the size structure of catch, gear regulations, and fishing-related employment. Examples of the latter are quantitative measures of social surplus, stock or ecosystem preservation, biodiversity, fishing sustainability, as well as the reduction in by-catch, discards, and of physical damages caused by fishing gear. The latter examples belong to the category of effects economic theory calls externalities; these ought to be integrated in quantitative analyses if unsustainable fishing regimes are to be detected and avoided.

To date, most attempts to quantify changes in utility arising from fishing have included only a small subset of traditional utility components (but see Dichmont *et al*. 2008 for an analysis of multiple utility components). Dankel *et al*. (2007) demonstrated how quantitative measures of stock preservation and fishing-related employment can be integrated into a utility function that also contains measures of yield and profit. Johnston *et al*. (2010) analysed how multicomponent utility functions can be used to optimize utility across heterogeneous groups of recreational fishers engaged in dynamic fishing behaviour. The utility components included in that study were based on minimum-size limits, licence costs, catch rates, average and maximum size of captured fish, and crowding among fishers.

In recognition of the potentially significant changes in utility that could result from FIE, some recent studies have attempted to quantify changes in utility brought about by demographic, plastic, and evolutionary changes (e.g. McAllister and Peterman 1992; Guttormsen *et al*. 2008; Okamoto *et al*. 2009; Eikeset 2010). In their theoretical bio-economic model, Guttormsen *et al*. (2008) studied the optimal long-term management of a renewable resource under harvest-induced selection. Their model shows that the optimal management regime depends not only on biological parameters of the resource, such as the productivity and growth rate of desirable vs. undesirable genotypes, but also on the considered discount rates (low discount rates favour a management regime that places more value on the long-term future state). Okamoto *et al*. (2009) showed how the objective of avoiding FIE can be used in a utility function to identify fishing regimes most suited to that purpose. Eikeset (2010) also specifically modelled FIE under different fishing scenarios and found that higher fishing mortality, causing FIE towards earlier maturation, eventually decreases economic yield in comparison with lower fishing mortality. Mollet (2010) used a model explicitly calibrated to historical life-history data and the rate of evolutionary response in North Sea flatfish to determine the evolutionary impact on traits by comparing models with and without evolution (Box 2). Furthermore, Mollet (2010) estimated the evolutionary impact on utility components such as yield and on reference points defined through maximum sustainable yield. Finally, when evaluating the outcome of different management scenarios on the aforementioned utility components, Mollet (2010) found that large fish should be protected to avoid undesired evolutionary impacts. Protecting large fish, however, trades off against short-term gains in yield, and this potentially generates conflicts of interest among stakeholders. Managers will thus have to balance long-term gains against short-term losses when maximizing yields over long time spans: EvoIA allows for transparency in the rationale behind management decisions.

An additional challenge arising when assessing the corresponding socioeconomic dynamics associated with fisheries is to account for the disparity of time horizons among stakeholders. For example, fishers often focus their interests on relatively short-term developments, whereas conservation groups usually advocate an emphasis on longer-term considerations. As we have already discussed above, attempts to capture such differences in the time horizons of stakeholders often involve the use of different discount rates, which convert future costs or benefits into different net present values that reflect the interests of different stakeholders. While this approach is meant to account for the different time preferences and opportunity costs of resource users, it has been argued that using market-based discount rates for managing natural resources is inherently problematic (e.g. Arndt 1993; Eikeset 2010). Thus, to achieve the sustainable use of fisheries resources, it may be appropriate to consider a discount rate of zero, or even to explore the effects of using a negative discount rate over a suitably chosen finite time horizon. The latter approach implies a particularly high regard for the well-being of future generations, by attributing a higher value to their benefits than to those of the current generation.

## Management-strategy evaluation

Management-strategy evaluation is a framework for assessing and comparing the differential merits of management strategies in the face of uncertainty (Smith *et al*. 1999; Bunnefeld *et al*. 2011). Naturally, methods already developed in the general context of MSE are valuable in the specific context of EvoIA. A management strategy is defined as a fully specified set of rules for determining management actions under a variety of circumstances. In its most general form, these rules include protocols for data collection and monitoring, as well as assessment procedures and decision rules for adjusting regulations (Dichmont *et al*. 2008). MSE is a simulation-based approach that can be used to quantitatively assess the performance of alternative management options with respect to specified management objectives (Smith 1993). Application of MSE to ecosystem management in general (Smith *et al*. 2007), and to fisheries management in particular (Dichmont *et al*. 2008), has been advocated as a robust method for comparing alternative management strategies in the face of multiple, and often conflicting, objectives. MSE requires the specification of three major elements: (i) a plausible operating model representing the considered fishery system including key uncertainties, (ii) a set of management strategies to be evaluated, and (iii) a performance metric corresponding to the objectives identified by decision-makers or stakeholders (Kell *et al*. 2006).

In the EvoIA framework, MSE methods can be used either for relatively simple tasks, such as examining whether a specific alternative management strategy should be adopted instead of a currently applied strategy, or for more complex tasks, such as selecting an optimal management strategy by evaluating a continuum of possible management options according to a given global utility function. MSE could thus offer a possible platform for embedding EvoIA in current practices for assessment and management by drawing on existing operating models and by extending these as necessary to cover the relevant ecological, evolutionary, and socioeconomic components. A particular appeal of interfacing EvoIA with MSE is the explicit treatment of uncertainty in MSE. Sources of uncertainty include observation error limiting the accuracy of monitoring efforts, parametric and structural uncertainty associated with operating models, process uncertainty resulting from fluctuations in the natural and socioeconomic subsystems, and implementation uncertainty involved in adopting and enforcing management measures. For example, uncertainty about estimated selection differentials or selection responses could be accommodated relatively easily by considering these quantities in terms of their distributions, while qualitatively different predictions about evolutionary dynamics could be treated as alternative hypotheses about the operating model.

## Discussion

Overexploited and collapsed fish stocks, poor recovery after fishing ceases, and altered interspecific interactions indicate that fisheries science and management are not accounting for all relevant factors that influence the dynamics of aquatic ecosystems (Francis *et al*. 2007). Evolutionary change is likely to be one such factor, but undoubtedly not the only one. We suggest that while FIE is certainly not the most important driver of the current fisheries crisis, it nevertheless deserves more attention, owing to its cumulative consequences and our still rather limited level of knowledge about its impacts. Currently, fisheries scientists and managers are facing uncertainty over the potential occurrence and implications of FIE in many stocks. EvoIA can help them to determine the prevalence and consequence of FIE, and to evaluate management measures accordingly (Jørgensen *et al*. 2007). Here, we have expanded upon the concept of EvoIA introduced by Jørgensen *et al*. (2007), outlining how an EvoIA can be structured, what functions it can fulfil, and which methods are available for its implementation.

The majority of methods highlighted in this paper are already in place. Yet, most of these methods have been developed in isolation and have been used for disparate purposes. In principle, these methods can be used to investigate any kind of environmental impact on marine systems, but we have here focused solely on the impacts of exploitation. EvoIA provides a framework for combining these methods towards the common purpose of assessing impacts of FIE on the utility of living aquatic resources. Nevertheless, it goes without saying that a continuous development of new methods will further strengthen the EvoIA approach. First, in addition to PMRNs (Dieckmann and Heino 2007) and common-garden experiments (Conover and Munch 2002; Reznick and Ghalambor 2005), other methods are necessary for controlling for environmental effects on phenotypes to convincingly show that observed phenotypic changes currently attributed to evolution are indeed most likely to have a genetic basis (Law 2000; Kuparinen and Merilä 2007). Even though genomic methods still cannot be used to predict complex phenotypic expressions of DNA variation, they are ultimately bound to offer valuable tools for analysing FIE (Naish and Hard 2008). The increasing power of high-throughput sequencing methods and the recent assembly of the Atlantic cod genome are promising steps in this direction (Star *et al*. 2011), and coupling genomic approaches with time series of historical samples will be particularly valuable (Poulsen *et al*. 2006; Nielsen *et al*. 2012). Second, estimating stock- and trait-specific selection differentials and then analysing their temporal correlations with fishing-mortality rates is another way of strengthening the evidence for FIE (Swain *et al*. 2007; Kendall *et al*. 2009). Third, to our knowledge, no methods have yet been developed for assessing possible evolutionary effects of fishing on behavioural traits in commercial fisheries (but see Philipp *et al*. 2009 for an example from recreational fishing), although there is considerable indirect and anecdotal evidence that behavioural evolution may well be widespread (Uusi-Heikkilä *et al*. 2008), preventing increases in catchability despite innovations in fishing technologies (Rijnsdorp *et al*. 2008). Fourth, improved quantitative and data-based tools are needed for assessing the differential evolutionary vulnerability of specific stocks. Naturally, the need for additional methodology must not delay the implementation of existing tools, as even small evolutionary changes can have surprisingly large effects on ecological processes in populations, communities, and ecosystems (Pelletier *et al*. 2009).

A possible application of EvoIA concerns the determination of reference points for fisheries management in a way that accounts for FIE (Hutchings 2009; ICES 2009; Mollet 2010). It has already been shown that reference points that fail to account for climate change may not be robust (e.g. Kell *et al*. 2005), which in turn may have implications for management advice. Analogously, reference points determined without accounting for potential FIE are likely to be biased, and those biases may grow over time (Enberg *et al*. 2010). Because reference points are key quantities in fisheries management – as illustrated by their pivotal role in harvest-control rules, especially in setting total allowable catches – hidden biases and trends are highly undesirable.

In many cases, fishing may be assumed to exert the main selection pressure on a fish stock (Heino 1998; Arlinghaus *et al*. 2009) and will therefore be the main selective force examined in an EvoIA. In other situations, additional external drivers, such as changes in climate or habitats (Carlson *et al*. 2007), selection on other life stages (Berkeley *et al*. 2004), and internal processes, such as sexual selection (Hutchings and Rowe 2008) and interspecific interactions (Gårdmark *et al*. 2003), can exert selection pressures on body size and other life-history traits that might be comparable in magnitude to those caused by fishing. These additional evolutionary forces can reinforce or oppose those underlying FIE (e.g. Dunlop *et al*. 2007) and should thus be accounted for in EvoIA as necessary. The flexibility of EvoIA, in terms of the diversity of available methods, facilitates such an inclusion of a number of important drivers of ecological and evolutionary processes.

Great complexity characterizes the possible impacts of FIE. In some cases, these impacts are desirable, such as when a declining age at maturation increases a stock's resilience to high fishing pressure (Heino 1998; Enberg *et al*. 2009). Without such FIE, more stocks might already have collapsed. However, life-history evolution often has undesirable consequences, and it is not easy to predict the ultimate extent of such evolutionary changes and their eventual implications (Jørgensen *et al*. 2007). Like climate change, anthropogenic evolution is caused by a multitude of distributed agents and has delayed effects on a global scale that accumulate over time. This unavoidably increases our uncertainty about long-term ecological changes associated with FIE and implies a certain risk of unexpected system-wide regime shifts caused by FIE. Through concerted scientific efforts across disciplines, climate-change science is currently rising to the challenge of predicting future trajectories of the physical system together with their socioeconomic implications (MacKenzie *et al*. 2007; Rijnsdorp *et al*. 2009). This achievement provides a promising precedent for tackling the complex ecological and socioeconomic impacts that can be expected from FIE.

The overlap between EvoIA and EAF-based management, in terms of goals and methods, is substantial (Francis *et al*. 2007): the way these two approaches complement each other is illustrated in Fig. 7. While a multispecies assessment might be challenging to achieve because of its complexity, it should nonetheless be the ultimate goal. However, a reasonable first step in considering the evolutionary consequences of fishing would be to implement single-species EvoIAs in systems where no EvoIAs have previously been made. Our recommendation to implement EvoIA is based on the recognition that evolution is an important ingredient of ecological dynamics (Pelletier *et al*. 2007; Carlson *et al*. 2011; Schoener 2011) because traits can evolve on timescales relevant for management. Owing to FIE, actors in the ecological theatre gradually change their roles and interactions over time. EAF-based management should therefore account for this possibility (FAO 2003). In the end, the relative contribution of FIE might turn out to be small compared with the ecological and environmental challenges already considered to be threatening sustainable fisheries (e.g. Andersen and Brander 2009). However, it is likely that specific management recommendations that decision-makers currently hesitate to implement will become even more compelling as knowledge about the effects of FIE grows through the implementation of EvoIA (Eikeset 2010). In many cases, evolutionary concerns align with the already-existing ecological concerns. In other cases, well-intentioned management focused on mitigating a particular ecological change may inadvertently induce undesired evolutionary change.

Undoubtedly, the EvoIA approach outlined here is highly complex and a full-scale EvoIA will be a challenging task. Beyond accounting for FIE in the estimates of demographics and sustainability, the effective incorporation into fisheries management will largely depend on the extent to which the various components proposed are taken up by fishery managers. Furthermore, because of the many building blocks – each with many parameters of which many are highly uncertain and inherently difficult to estimate – it can be easy to dismiss this approach as a purely academic exercise without practical value. However, the complicated characteristics of ecological, evolutionary and socio-economic processes do not lend themselves well to simplified analyses. Thus, the EAF mandates that the scientific basis for management decision rely on analyses that are as complicated as necessary to incorporate all relevant factors. Moreover, the fact that we, in many cases, may have to rely on models including a high level of uncertainty should in any case not be an excuse for inaction. As a start, progressively building and extending assessment models by including evolutionary thinking into practices will be more realistic than an immediate implementation of the whole framework. However, because there is a strong need for immediate operational advice, we have, in Table 1, summarized general expectations for FIE for two types of selectivity patterns, as well as possible mitigative actions. While we are reluctant to provide explicit advice on how to reduce the potential for FIE when relatively few stocks have been investigated, we can observe that a dome-shaped selection pattern almost always is beneficial for reducing FIE. See also Garcia *et al*. (2012) for general advice on balanced harvesting.

Improved assessment of the evolutionary impacts of fishing can lead to better management practices and more accurate predictions of stock dynamics and ecosystem effects. Failure to investigate the presence of, and account for, FIE in stock assessments, management advice, and policy making may exacerbate the negative consequences of phenotypic changes already commonly observed across the fish stocks we aim to sustain.

## References

[b1] Aas Ø, Haider W, Hunt L (2000). Angler responses to potential harvest regulations in a Norwegian sport fishery: a conjoint-based choice modeling approach. North American Journal of Fisheries Management.

[b2] Allendorf FW, England PR, Luikart G, Ritchie PA, Ryman N (2008). Genetic effects of harvest on wild animal populations. Trends in Ecology and Evolution.

[b3] Andersen KH, Brander K (2009). Expected rate of fisheries-induced evolution is slow. Proceedings of the National Academy of Sciences of the United States of America.

[b4] Anderson CNK, Hsieh CH, Sandin SA (2008). Why fishing magnifies fluctuations in fish abundance. Nature.

[b5] Arlinghaus R, Matsumura S, Dieckmann U (2009). Quantifying selection differentials caused by recreational fishing: development of modeling framework and application to reproductive investment in pike (*Esox lucius*. Evolutionary Applications.

[b6] Arlinghaus R, Matsumura S, Dieckmann U (2010). The conservation and fishery benefits of protecting large pike (*Esox lucius* L.) by harvest regulations in recreational fishing. Biological Conservation.

[b7] Arndt HW (1993). Sustainable development and the discount rate. Economic Development and Cultural Change.

[b8] Askey PJ, Richards SA, Post JR, Parkinson EA (2006). Linking angling catch rates and fish learning under catch-and-release regulations. North American Journal of Fisheries Management.

[b9] Balmford A, Bruner A, Cooper P (2002). Economic reasons for conserving wild nature. Science.

[b10] Bannock G, Baxter RE, Davis E (2003). Dictionary of Economics.

[b11] Baskett ML, Levin SA, Gaines SD, Dushoff J (2005). Marine reserve design and the evolution of size at maturation in harvested fish. Ecological Applications.

[b12] Baulier L (2009). Evolutionary and statistical modeling of life-time schedules of energy allocation in Atlantic herring and cod.

[b13] BenDor T, Scheffran J, Hannon B (2009). Ecological and economic sustainability in fishery management: a multi-agent model for understanding competition and cooperation. Ecological Economics.

[b14] Berkeley SA, Chapman C, Sogard SM (2004). Maternal age as a determinant of larval growth and survival in a marine fish, *Sebastes melanops*. Ecology.

[b15] Beverton RJH, Holt SJ (1957). On the dynamics of exploited fish populations. Fishery Investigations Series II (Marine Fisheries, Great Britain; Ministry of Agriculture, Fisheries and Food, London, UK).

[b16] Biro PA, Post JR (2008). Rapid depletion of genotypes with fast growth and bold personality traits from harvested fish populations. Proceedings of the National Academy of Sciences of the United States of America.

[b17] Boukal DS, Dunlop ES, Heino M, Dieckmann U (2008).

[b18] Bradshaw CJA, Mollet HF, Meekan MG (2007). Inferring population trends for the world's largest fish from mark-recapture estimates of survival. Journal of Animal Ecology.

[b19] Branch TA, Hilborn R, Haynie AC (2006). Fleet dynamics and fishermen behavior: lessons for fisheries managers. Canadian Journal of Fisheries and Aquatic Sciences.

[b20] Bunnefeld N, Hoshino E, Milner-Gulland EJ (2011). Management strategy evaluation: a powerful tool for conservation?. Trends in Ecology and Evolution.

[b21] Cadrin SX, Pastoors MA (2008). Precautionary harvest policies and the uncertainty paradox. Fisheries Research.

[b22] Calhoun C (2002). Dictionary of the Social Sciences.

[b23] Carlson SM, Edeline E, Vøllestad LA (2007). Four decades of opposing natural and human-induced artificial selection acting on Windermere pike (*Esox lucius*. Ecology Letters.

[b24] Carlson SM, Quinn TP, Hendry AP (2011). Eco-evolutionary dynamics in Pacific salmon. Heredity.

[b25] Carpenter SR, Brock WA, Ludwig D (2007). Appropriate discounting leads to forward-looking ecosystem management. Ecological Research.

[b26] Charles A (2001). Sustainable Fishery Systems.

[b27] Choi JS, Frank KT, Leggett WC, Drinkwater K (2004). Transition to an alternate state in a continental shelf ecosystem. Canadian Journal of Fisheries and Aquatic Sciences.

[b28] Christensen A, Andersen K (2011). General classification of maturation reaction-norm shape from size-based processes. Bulletin of Mathematical Biology.

[b29] Conover DO, Munch SB (2002). Sustaining fisheries yields over evolutionary time scales. Science.

[b30] Conover DO, Munch SB, Arnott SA (2009). Reversal of evolutionary downsizing caused by selective harvest of large fish. Proceedings of the Royal Society B: Biological Sciences.

[b31] Conrad M (1983). Adaptability: The Significance of Variability from Molecular to Ecosystem.

[b32] Costanza R (2008). Ecosystem services: multiple classification systems are needed. Biological Conservation.

[b33] Costanza R, d'Arge R, de Groot R (1997). The value of the world's ecosystem services and natural capital. Nature.

[b34] Daily GC (1997). Nature's Services: Societal Dependence on Natural Ecosystems.

[b35] Dankel DJ (2009). Building blocks of sustainability in marine fisheries management. Stakeholders, objectives, and strategies.

[b36] Dankel DJ, Heino M, Dieckmann U (2007).

[b37] Darimont CT, Carlson SM, Kinnison MT, Paquet PC, Reimchen TE, Wilmers CC (2009). Human predators outpace other agents of trait change in the wild. Proceedings of the National Academy of Sciences of the United States of America.

[b38] Daskalov GM, Grishin AN, Rodionov S, Mihneva V (2007). Trophic cascades triggered by overfishing reveal possible mechanisms of ecosystem regime shifts. Proceedings of the National Academy of Sciences of the United States of America.

[b39] Dichmont CM, Deng A, Punt AE (2008). Beyond biological performance measures in management strategy evaluation: bringing in economics and the effects of trawling on the benthos. Fisheries Research.

[b40] Dieckmann U, Heino M (2007). Probabilistic maturation reaction norms: their history, strengths, and limitations. Marine Ecology-Progress Series.

[b41] Dorow M, Beardmore B, Haider W, Arlinghaus R (2010). Winners and losers of conservation policies for European eel, *Anguilla anguilla*: an economic welfare analysis for differently specialised eel anglers. Fisheries Management and Ecology.

[b42] Dunlop ES, Shuter BJ, Dieckmann U (2007). Demographic and evolutionary consequences of selective mortality: predictions from an eco-genetic model for smallmouth bass. Transactions of the American Fisheries Society.

[b43] Dunlop ES, Heino M, Dieckmann U (2009a). Eco-genetic modeling of contemporary life-history evolution. Ecological Applications.

[b44] Dunlop ES, Baskett ML, Heino M, Dieckmann U (2009b). Propensity of marine reserves to reduce the evolutionary effects of fishing in a migratory species. Evolutionary Applications.

[b45] Edeline E, Carlson SM, Stige LC (2007). Trait changes in a harvested population are driven by a dynamic tug-of-war between natural and harvest selection. Proceedings of the National Academy of Sciences of the United States of America.

[b46] Eikeset AM (2010). The ecological and evolutionary effects of harvesting Northeast Arctic cod – insights from economics and implications for management.

[b47] Eldridge WH (2007). Human impacts on the genetic diversity of Pacific salmon through hatcheries and harvest.

[b48] Enberg K, Jørgensen C, Dunlop ES, Heino M, Dieckmann U (2009). Implications of fisheries-induced evolution for stock rebuilding and recovery. Evolutionary Applications.

[b49] Enberg K, Jørgensen C, Mangel M (2010). Fishing-induced evolution and changing reproductive ecology of fish: the evolution of steepness. Canadian Journal of Fisheries and Aquatic Sciences.

[b50] Enberg K, Jorgensen C, Dunlop ES (2012). Fishing-induced evolution of growth: concepts, mechanisms and the empirical evidence. Marine Ecology.

[b51] Engelhard GH, Heino M (2004). Maturity changes in Norwegian spring-spawning herring *Clupea harengus*: compensatory or evolutionary responses?. Marine Ecology-Progress Series.

[b52] Ernande B, Dieckmann U, Heino M (2004). Adaptive changes in harvested populations: plasticity and evolution of age and size at maturation. Proceedings of the Royal Society B: Biological Sciences.

[b53] FAO Fisheries Department (2003). The ecosystem approach to fisheries. FAO Technical Guidelines for Responsible Fisheries.

[b54] Francis RC, Hixon MA, Clarke ME, Murawski SA, Ralston S (2007). Ten commandments for ecosystem-based fisheries scientists. Fisheries.

[b55] Frank KT, Petrie B, Choi JS, Leggett WC (2005). Trophic cascades in a formerly cod-dominated ecosystem. Science.

[b56] Frank KT, Petrie B, Fisher JAD, Leggett WC (2011). Transient dynamics of an altered large marine ecosystem. Nature.

[b57] Garcia SM, Cochrane KL (2005). Ecosystem approach to fisheries: a review of implementation guidelines. ICES Journal of Marine Science.

[b58] Garcia SM, Kolding J, Rice J (2012). Reconsidering the consequences of selective fisheries. Science.

[b59] Gårdmark A, Dieckmann U, Lundberg P (2003). Life-history evolution in harvested populations: the role of natural predation. Evolutionary Ecology Research.

[b60] Goldsworthy SD, He X, Tuck GN, Lewis M, Williams R (2001). Trophic interactions between the Patagonian toothfish, its fishery, and seals and seabirds around Macquarie Island. Marine Ecology-Progress Series.

[b61] Grift RE, Rijnsdorp AD, Barot S, Heino M, Dieckmann U (2003). Fisheries-induced trends in reaction norms for maturation in North Sea plaice. Marine Ecology-Progress Series.

[b62] Grift RE, Heino M, Rijnsdorp AD, Kraak SBM, Dieckmann U (2007). Three-dimensional maturation reaction norms for North Sea plaice. Marine Ecology-Progress Series.

[b63] Guttormsen AG, Kristofersson D, Nævdal E (2008). Optimal management of renewable resources with Darwinian selection induced by harvesting. Journal of Environmental Economics and Management.

[b64] Hard JJ, Gross MR, Heino M (2008). Evolutionary consequences of fishing and their implications for salmon. Evolutionary Applications.

[b65] Hauser L, Adcock GJ, Smith PJ, Ramirez JHB, Carvalho GR (2002). Loss of microsatellite diversity and low effective population size in an overexploited population of New Zealand snapper (*Pagrus auratus*. Proceedings of the National Academy of Sciences of the United States of America.

[b66] Hein L, van Koppen K, de Groot RS, Vanierland E (2006). Spatial scales, stakeholders and the valuation of ecosystem services. Ecological Economics.

[b67] Heino M (1998). Management of evolving fish stocks. Canadian Journal of Fisheries and Aquatic Sciences.

[b68] Heino M, Godø OR (2002). Fisheries-induced selection pressures in the context of sustainable fisheries. Bulletin of Marine Science.

[b69] Heino M, Dieckmann U, Godø OR (2002).

[b70] Hensher DA, Rose GM, Greene WH (2005). Applied Choice Analysis – A Primer.

[b71] Hiddink JG, Jennings S, Kaiser MJ, Queiros AM, Duplisea DE, Piet GJ (2006). Cumulative impacts of seabed trawl disturbance on benthic biomass, production, and species richness in different habitats. Canadian Journal of Fisheries and Aquatic Sciences.

[b72] Hilborn R (2007). Defining success in fisheries and conflicts in objectives. Marine PoIicy.

[b73] Hilborn R, Minte-Vera CV (2008). Fisheries-induced changes in growth rates in marine fisheries: are they significant?. Bulletin of Marine Science.

[b74] Holmes SJ, Wright PJ, Fryer RJ (2008). Evidence from survey data for regional variability in cod dynamics in the North Sea and West of Scotland. ICES Journal of Marine Science.

[b75] Hsieh CH, Yamauchi A, Nakazawa T, Wang WF (2010). Fishing effects on age and spatial structures undermine population stability of fishes. Aquatic Sciences.

[b76] Huse G, Railsback S, Ferno A (2002). Modelling changes in migration pattern of herring: collective behaviour and numerical domination. Journal of Fish Biology.

[b77] Hutchings JA (2009). Avoidance of fisheries-induced evolution: management implications for catch selectivity and limit reference points. Evolutionary Applications.

[b78] Hutchings JA, Myers RA (1994). What can be learned from the collapse of a renewable resource? Atlantic cod, *Gadus morhua*, of Newfoundland and Labrador. Canadian Journal of Fisheries and Aquatic Sciences.

[b79] Hutchings JA, Rowe S (2008). Consequences of sexual selection for fisheries-induced evolution: an exploratory analysis. Evolutionary Applications.

[b80] Hutchinson WF (2008). The dangers of ignoring stock complexity in fishery management: the case of the North Sea cod. Biology Letters.

[b81] Hutchinson WF, van Oosterhout C, Rogers SI, Carvalho GR (2003). Temporal analysis of archived samples indicates marked genetic changes in declining North Sea cod (*Gadus morhua*. Proceedings of the Royal Society B: Biological Sciences.

[b82] ICES (2007).

[b83] ICES (2009).

[b84] Jackson JBC, Kirby MX, Berger WH, Hixon MA (2001). Historical overfishing and the recent collapse of coastal ecosystems. Science.

[b85] Johnson DW, Christie MR, Moye J, Hixon MA (2011). Genetic correlations between adults and larvae in a marine fish: potential effects of fishery selection on population replenishment. Evolutionary Applications.

[b86] Johnston F, Arlinghaus R, Dieckmann U (2010). Diversity and complexity of angler behaviour drive socially optimal input and output regulations in a bioeconomic recreational fisheries model. Canadian Journal of Fisheries and Aquatic Sciences.

[b87] Jørgensen T (1990). Long-term changes in age at sexual maturity of Northeast Arctic cod (*Gadus morhua* L.). Journal du Conseil International pour l'Exploration de la Mer.

[b88] Jørgensen C, Enberg K, Dunlop ES (2007). Managing evolving fish stocks. Science.

[b89] Jørgensen C, Dunlop ES, Opdal AF, Fiksen Ø (2008). The evolution of spawning migrations: state dependence and fishing-induced changes. Ecology.

[b90] Jørgensen C, Ernande B, Fiksen Ø (2009). Size-selective fishing gear and life history evolution in the Northeast Arctic cod. Evolutionary Applications.

[b91] Kaiser MJ, Edwards-Jones G (2006). The role of ecolabeling in fisheries management and conservation. Conservation Biology.

[b92] Kell LT, Pilling GM, O'Brien CA (2005). Implications of climate change for the management of North Sea cod (*Gadus morhua*. ICES Journal of Marine Science.

[b93] Kell LT, Pilling GM, Kirkwood GP (2006). An evaluation of multi-annual management strategies for ICES roundfish stocks. ICES Journal of Marine Science.

[b94] Kendall NW, Hard JJ, Quinn TP (2009). Quantifying six decades of fishery selection for size and age at maturity in sockeye salmon. Evolutionary Applications.

[b95] Kinnison MT, Quinn TP, Unwin MJ (2011). Correlated contemporary evolution of life history traits in New Zealand Chinook salmon, *Oncorhynchus tshawytscha*. Heredity.

[b96] van Kooten T, Andersson J, Byström P, Persson L, de Roos AM (2010). Size at hatching determines population dynamics and response to harvesting in cannibalistic fish. Canadian Journal of Fisheries and Aquatic Sciences.

[b97] Kuparinen A, Merilä J (2007). Detecting and managing fisheries-induced evolution. Trends in Ecology and Evolution.

[b98] Kuparinen A, Kuikka S, Merilä J (2009). Estimating fisheries-induced selection: traditional gear selectivity research meets fisheries-induced evolution. Evolutionary Applications.

[b99] Kuparinen A, Cano J, Loehr J, Herczeg G, Gonda A, Merilä J (2011). Fish age at maturation is influenced by temperature independently of growth. Oecologia.

[b100] Larkin PA (1977). Epitaph for concept of maximum sustained yield. Transactions of the American Fisheries Society.

[b101] Law R (2000). Fishing, selection and phenotypic evolution. ICES Journal of Marine Science.

[b102] Law R, Grey DR (1989). Evolution of yields from populations with age-specific cropping. Evolutionary Ecology.

[b103] Law R, Rowell CA, Stokes TK, McGlade JM, Law R (1993). Cohort-structured populations, selection responses, and exploitation of the North Sea cod. The Exploitation of Evolving Resources.

[b104] MacKenzie BR, Gislason H, Möllmann C, Köster FW (2007). Impact of 21st century climate change on the Baltic Sea fish community and fisheries. Global Change Biology.

[b105] Marty L, Dieckmann U, Rochet M-J, Ernande B (2011). Impact of environmental covariation in growth and mortality on evolving maturation reaction norms. American Naturalist.

[b106] Matsuda H, Abrams PA (2004). Effects of predator-prey interactions and adaptive change on sustainable yield. Canadian Journal of Fisheries and Aquatic Sciences.

[b107] Matsumura S, Arlinghaus R, Dieckmann U (2011). Assessing evolutionary consequences of size-selective recreational fishing on multiple life-history traits, with an application to northern pike (*Esox lucius*. Evolutionary Ecology.

[b108] McAllister MK, Peterman RM (1992). Decision analysis of a large-scale fishing experiment designed to test for a genetic effect of size-selective fishing on British Columbia pink salmon (*Oncorhynchus-gorbuscha*. Canadian Journal of Fisheries and Aquatic Sciences.

[b109] McFadden D, Zarembka P (1974). Conditional logit analysis of qualitative choice behavior. Frontiers in Econometrics.

[b110] Millennium Ecosystem Assessment (2003). Ecosystems and Human Well-Being. A Framework for Assessment.

[b111] Mollet F (2010). Evolutionary effects of fishing and implications for sustainable management: a case study of North Sea plaice and sole.

[b112] Mollet FM, Kraak SMB, Rijnsdorp AD (2007). Fisheries-induced evolutionary changes in maturation reaction norms in North Sea sole *Solea solea*. Marine Ecology-Progress Series.

[b113] Mollet FM, Poos JJ, Dieckmann U, Rijnsdorp AD (2010). Evolutionary impact assessment of commercial fisheries: a case study of North Sea flatfish fisheries management. Evolutionary effects of fishing and implications for sustainable management: a case study of North Sea plaice and sole.

[b114] Morita K, Fukuwaka MA (2006). Does size matter most? The effect of growth history on probabilistic reaction norm for salmon maturation. Evolution.

[b115] Naish KA, Hard JJ (2008). Bridging the gap between the genotype and the phenotype: linking genetic variation, selection and adaptation in fishes. Fish and Fisheries.

[b116] Nielsen EE, Cariani A, Aoidh EM (2012). Gene-associated markers provide tools for tackling illegal fishing and false eco-certification. Nature Communications.

[b117] Nusslé S, Bornand CN, Wedekind C (2008). Fishery-induced selection on an Alpine whitefish: quantifying genetic and environmental effects on individual growth rate. Evolutionary Applications.

[b118] Okamoto KW, Whitlock R, Magnan P, Dieckmann U (2009). Mitigating fisheries-induced evolution in lacustrine brook charr (*Salvelinus fontinalis*) in southern Quebec, Canada. Evolutionary Applications.

[b119] Olsen EM, Moland E (2011). Fitness landscape of Atlantic cod shaped by harvest selection and natural selection. Evolutionary Ecology.

[b120] Overholtz WJ (2002). The Gulf of Maine-Georges Bank Atlantic herring (*Clupea harengus)*: spatial pattern analysis of the collapse and recovery of a large marine fish complex. Fisheries Research.

[b121] Palkovacs EP, Wasserman BA, Kinnison MT (2011). Eco-evolutionary trophic dynamics: loss of top predators drives trophic evolution and ecology of prey. PLoS ONE.

[b122] Palkovacs EP, Kinnison MT, Correa C, Dalton CM, Hendry AP (2012). Fates beyond traits: ecological consequences of human-induced trait change. Evolutionary Applications.

[b123] Parsons DM (2011). A fisheries perspective of behavioural variability: differences in movement behaviour and extraction rate of an exploited sparid, snapper (*Pagrus auratus*. Canadian Journal of Fisheries and Aquatic Sciences.

[b124] Pelletier F, Clutton-Brock T, Pemberton J, Tuljapurkar S, Coulson T (2007). The evolutionary demography of ecological change: linking trait variation and population growth. Science.

[b125] Pelletier F, Garant D, Hendry AP (2009). Eco-evolutionary dynamics. Philosophical Transactions of the Royal Society B: Biological Sciences.

[b126] Persson L, Amundsen PA, De Roos AM, Klemetsen A, Knudsen R, Raul Primicerio (2007). Culling prey promotes predator recovery – alternative states in a whole-lake experiment. Science.

[b127] Philipp DP, Cooke SJ, Claussen JE, Koppelman J, Suski C, Burkett D (2009). Selection for vulnerability to angling in largemouth bass. Transactions of the American Fisheries Society.

[b128] Poulsen NA, Nielsen EE, Schierup MH, Loeschcke V, Grønkjær P (2006). Long-term stability and effective population size in North Sea and Baltic Sea cod (*Gadus morhua*. Molecular Ecology.

[b129] Prodanov K, Mikhaylov K, Daskalov G (1995). On the problem of commercial fishing influence on the abundance and biomass of mackerel *Scomber scombrus* L., bonito *Sarda sarda* L. and bluefish *Pomatomus saltatrix* L. Ecological Problems and Economical Prospects.

[b130] Raat AJP (1985). Analysis of angling vulnerability of common carp, *Cyprinus carp* L, in catch-and-release angling in ponds. Aquaculture Research.

[b131] Restrepo VR (1999). Providing scientific advice to implement the precautionary approach under the Magnuson-Steven Fishery Conservation and Management Act. Proceedings of the Fifth National NMFS Stock Assessment Workshop.

[b132] Reznick DN, Ghalambor CK (2005). Can commercial fishing cause evolution? Answers from guppies (*Poecilia reticulata*. Canadian Journal of Fisheries and Aquatic Sciences.

[b133] Reznick DN, Stokes TK, McGlade JM, Law R (1993). Norms of reaction in fishes. The Exploitation of Evolving Resources.

[b134] Rijnsdorp AD, Grift RE, Kraak SBM (2005). Fisheries-induced adaptive change in reproductive investment in North Sea plaice (*Pleuronectes platessa*)?. Canadian Journal of Fisheries and Aquatic Sciences.

[b135] Rijnsdorp AD, Poos JJ, Quirijns FJ, HilleRisLambers R, De Wilde JW, Den Heijer WM (2008). The arms race between fishers. Journal of Sea Research.

[b136] Rijnsdorp AD, Peck MA, Engelhard GH, Möllmann C, Pinnegar JK (2009). Resolving the effect of climate change on fish populations. ICES Journal of Marine Science.

[b137] Rijnsdorp A, van Overzee H, Poos J (2012). Ecological and economic trade-offs in the management of mixed fisheries: a case study of spawning closures in flatfish fisheries. Marine Ecology Progress Series.

[b138] de Roos AM, Boukal DS, Persson L (2006). Evolutionary regime shifts in age and size at maturation of exploited fish stocks. Proceedings of the Royal Society B: Biological Sciences.

[b139] Ruzzante DE, Mariani S, Bekkevold D (2006). Biocomplexity in a highly migratory pelagic marine fish, Atlantic herring. Journal of Fish Biology.

[b140] Salas S, Gaertner D (2004). The behavioural dynamics of fishers: management implications. Fish and Fisheries.

[b141] Sattar SA, Jørgensen C, Fiksen Ø (2008). Fisheries-induced evolution of energy and sex allocation. Bulletin of Marine Science.

[b142] Saura M, Morán P, Brotherstone S, Caballero A, Alvarez J, Villanueva B (2010). Predictions of response to selection caused by angling in a wild population of Atlantic salmon (*Salmo salar)*. Freshwater Biology.

[b143] Schoener TW (2011). The Newest Synthesis: understanding the interplay of evolutionary and ecological dynamics. Science.

[b144] Shackell NL, Frank KT, Fisher JAD, Petrie B, Leggett WC (2010). Decline in top predator body size and changing climate alter trophic structure in an oceanic ecosystem. Proceedings of the Royal Society B: Biological Sciences.

[b145] Sharpe DMT, Hendry AP (2009). Life history change in commercially exploited fish stocks: an analysis of trends across studies. Evolutionary Applications.

[b146] Sinclair AF, Swain DP, Hanson JM (2002). Measuring changes in the direction and magnitude of size-selective mortality in a commercial fish population. Canadian Journal of Fisheries and Aquatic Sciences.

[b147] Smith ADM (1993).

[b148] Smith ADM, Sainsbury KJ, Stevens RA (1999). Implementing effective fisheries-management systems – management strategy evaluation and the Australian partnership approach. ICES Journal of Marine Science.

[b149] Smith ADM, Fulton EJ, Hobday AJ, Smith C, Shoulder P (2007). Scientific tools to support the practical implementation of ecosystem-based fisheries management. ICES Journal of Marine Science.

[b150] Star B, Nederbragt AJ, Jentoft S (2011). The genome sequence of Atlantic cod reveals a unique immune system. Nature.

[b151] Swain DP, Sinclair AF, Hanson JM (2007). Evolutionary response to size-selective mortality in an exploited fish population. Proceedings of the Royal Society B: Biological Sciences.

[b152] Symes D, Phillipson J (2009). Whatever became of social objectives in fisheries policy?. Fisheries Research.

[b153] Thériault V, Dunlop ES, Dieckmann U, Bernatchez L, Dodson JJ (2008). The impact of fishing-induced mortality on the evolution of alternative life-history tactics in brook charr. Evolutionary Applications.

[b154] Therkildsen NO, Nielsen EE, Swain DP, Pedersen JS (2010). Large effective population size and temporal genetic stability in Atlantic cod (*Gadus morhua*) in the southern Gulf of St. Lawrence. Canadian Journal of Fisheries and Aquatic Sciences.

[b155] Toresen R, Østvedt OJ (2000). Variation in abundance of Norwegian spring spawning herring (*Clupea harengus,* Clupeidae) throughout the 20th century and the influence of climatic fluctuations. Fish and Fisheries.

[b156] UN (1992).

[b157] Urbach D, Cotton S (2008). Comment: on the consequences of sexual selection for fisheries-induced evolution. Evolutionary Applications.

[b158] Uusi-Heikkilä S, Wolter C, Klefoth T, Arlinghaus A (2008). A behavioral perspective on fishing-induced evolution. Trends in Ecology and Evolution.

[b159] Uusi-Heikkilä S, Kuparinen A, Wolter C, O'Toole AC, Arlinghaus R (2011). Experimental assessment of the probabilistic maturation reaction norm: condition matters. Proceedings of the Royal Society B: Biological Sciences.

[b160] Vainikka A, Hyvärinen P (2012). Ecologically and evolutionarily sustainable fishing of the pikeperch *Sander lucioperca*: Lake Oulujärvi as an example. Fisheries Research.

[b161] Vainikka A, Gårdmark A, Bland B, Hjelm J (2009). Two- and three-dimensional maturation reaction norms for the eastern Baltic cod, *Gadus morhua*. Ices Journal of Marine Science.

[b162] Venturelli PA, Shuter BJ, Murphy CA (2009). Evidence for harvest-induced maternal influences on the reproductive rates of fish populations. Proceedings of the Royal Society B: Biological Sciences.

[b163] Wallace KJ (2007). Classification of ecosystem services: problems and solutions. Biological Conservation.

[b164] Walsh MR, Munch SB, Chiba S, Conover DO (2006). Maladaptive changes in multiple traits caused by fishing: impediments to population recovery. Ecology Letters.

[b165] Walters CJ, Martell SJD (2004). Fisheries Ecology and Management.

[b166] Wang HY, Höök TO (2009). Eco-genetic model to explore fishing-induced ecological and evolutionary effects on growth and maturation schedules. Evolutionary Applications.

[b167] Watling L, Norse EA (1998). Disturbance of the seabed by mobile fishing gear: a comparison to forest clearcutting. Conservation Biology.

[b168] Wattage P, Mardle S, Pascoe S (2005). Evaluation of the importance of fisheries management objectives using choice-experiments. Ecological Economics.

[b169] Wohlfarth G, Moav R, Hulata G (1975). Genetic variation in seine escapability of common carp. Aquaculture.

[b170] Yoneda M, Wright PJ (2004). Temporal and spatial variation in reproductive investment of Atlantic cod *Gadus morhua* in the northern North Sea and Scottish west coast. Marine Ecology-Progress Series.

